# Plant-Derived Epi-Nutraceuticals as Potential Broad-Spectrum Anti-Viral Agents

**DOI:** 10.3390/nu15224719

**Published:** 2023-11-08

**Authors:** Rosita Gabbianelli, Ehud Shahar, Gaia de Simone, Chiara Rucci, Laura Bordoni, Giulia Feliziani, Fanrui Zhao, Marta Ferrati, Filippo Maggi, Eleonora Spinozzi, Jamal Mahajna

**Affiliations:** 1 Unit of Molecular Biology and Nutrigenomics, University of Camerino, Via Madonna delle Carceri, 62032 Camerino, Italy; rosita.gabbianelli@unicam.it (R.G.); gaia.desimone@unicam.it (G.d.S.); laura.bordoni@unicam.it (L.B.); giulia.feliziani@studenti.unicam.it (G.F.); fanrui.zhao@unicam.it (F.Z.); 2 Department of Nutrition and Natural Products, Migal—Galilee Research Institute, Kiryat Shmona 11016, Israel; ehudsha@migal.org.il; 3 Department of Biotechnology, Tel-Hai College, Kiryat Shmona 1220800, Israel; 4 Chemistry Interdisciplinary Project (ChIP) Research Centre, School of Pharmacy, University of Camerino, Via Madonna delle Carceri, 62032 Camerino, Italy; marta.ferrati@unicam.it (M.F.); filippo.maggi@unicam.it (F.M.); eleonora.spinozzi@unicam.it (E.S.)

**Keywords:** plant-derived substances, epigenetic modifications, virus, broad-spectrum anti-viral

## Abstract

Although the COVID-19 pandemic appears to be diminishing, the emergence of SARS-CoV-2 variants represents a threat to humans due to their inherent transmissibility, immunological evasion, virulence, and invulnerability to existing therapies. The COVID-19 pandemic affected more than 500 million people and caused over 6 million deaths. Vaccines are essential, but in circumstances in which vaccination is not accessible or in individuals with compromised immune systems, drugs can provide additional protection. Targeting host signaling pathways is recommended due to their genomic stability and resistance barriers. Moreover, targeting host factors allows us to develop compounds that are effective against different viral variants as well as against newly emerging virus strains. In recent years, the globe has experienced climate change, which may contribute to the emergence and spread of infectious diseases through a variety of factors. Warmer temperatures and changing precipitation patterns can increase the geographic range of disease-carrying vectors, increasing the risk of diseases spreading to new areas. Climate change may also affect vector behavior, leading to a longer breeding season and more breeding sites for disease vectors. Climate change may also disrupt ecosystems, bringing humans closer to wildlife that transmits zoonotic diseases. All the above factors may accelerate the emergence of new viral epidemics. Plant-derived products, which have been used in traditional medicine for treating pathological conditions, offer structurally novel therapeutic compounds, including those with anti-viral activity. In addition, plant-derived bioactive substances might serve as the ideal basis for developing sustainable/efficient/cost-effective anti-viral alternatives. Interest in herbal antiviral products has increased. More than 50% of approved drugs originate from herbal sources. Plant-derived compounds offer diverse structures and bioactive molecules that are candidates for new drug development. Combining these therapies with conventional drugs could improve patient outcomes. Epigenetics modifications in the genome can affect gene expression without altering DNA sequences. Host cells can use epigenetic gene regulation as a mechanism to silence incoming viral DNA molecules, while viruses recruit cellular epitranscriptomic (covalent modifications of RNAs) modifiers to increase the translational efficiency and transcript stability of viral transcripts to enhance viral gene expression and replication. Moreover, viruses manipulate host cells’ epigenetic machinery to ensure productive viral infections. Environmental factors, such as natural products, may influence epigenetic modifications. In this review, we explore the potential of plant-derived substances as epigenetic modifiers for broad-spectrum anti-viral activity, reviewing their modulation processes and anti-viral effects on DNA and RNA viruses, as well as addressing future research objectives in this rapidly emerging field.

## 1. Introduction

### 1.1. The Viral Life Cycle

Viruses represent a special category of organisms as they are unable to carry out essential life processes such as metabolism and reproduction, making them completely dependent on host cells for replication and rendering them obligate intracellular parasites [1]. The basic architecture of a virus includes a genome enclosed in a protective protein envelope called a capsid [2]. The viral genome can consist of either DNA or RNA oligonucleotides, which can be either single-stranded or double-stranded. The type of genetic material a virus carries has significant implications for its replication strategy and overall life cycle. For example, DNA viruses (e.g., herpes simplex virus (HSV)) generally replicate in the nucleus of the host cell using the host’s DNA and RNA synthesis machinery. In contrast, RNA viruses (such as influenza A virus (IAV)) usually replicate in the cytoplasm and therefore must encode or carry their own RNA polymerase to replicate their genome. In addition, some RNA viruses, called retroviruses (such as human immunodeficiency virus (HIV)), can reverse-transcribe their RNA genome into DNA, which is then integrated into the host genome—a unique feature of this category of viruses [3].

The viral genome encodes structural and nonstructural proteins. Structural proteins are self-assembled after their synthesis to form a highly structured, typically geometrically symmetric capsid [4]. Capsid proteins can assemble into a variety of different structures depending on the virus, but most capsids fall into one of two categories: icosahedral or helical [5,6]. Additional complex layers are added in certain viruses that have an additional lipid bilayer, called the envelope, which is extracted from the host cell membrane during viral assembly and release [7]. Viral nonstructural proteins (NSPs) encoded by the viral genome are not part of the viral particle or virion but play a key role in the viral life cycle. These proteins contribute to a variety of functions, including viral replication and assembly [8,9], the modulation of host immune responses [10], and the manipulation of the cellular environment to favor viral reproduction [11,12].

Viruses can cause a wide range of diseases, from trivial to life-threatening. They are the causative agents of common illnesses such as the common cold (rhinoviruses) and IVA, but they can also cause serious diseases such as HIV/acquired immunodeficiency syndrome (AIDS), Ebola hemorrhagic fever, and COVID-19 disease caused by SARS-CoV-2 [13,14]. The manifestation of these diseases depends largely on the host’s immune response to viral infection and the extent of tissue damage caused by viral replication and cell death.

Some viruses are exclusively adapted to humans, whereas others, known as zoonotic viruses, can also infect animal hosts [15]. Viruses such as IVA, measles, and SARS-CoV-2 are transmitted through the respiratory tract by droplets that are emitted when an infected person coughs, sneezes, or talks and can then be inhaled by a susceptible person, resulting in infection [16,17]. Other viruses, such as norovirus and rotavirus, are transmitted orally by the ingestion of contaminated food or water or by direct contact with the feces of an infected person [18]. Direct contact can result in viral transmission, either between people, as with HIV and hepatitis B (HBV) and C (HCV), which are transmitted through sexual contact, needle sharing, or at birth [19,20], or from animals to people, as exemplified by rabies virus, which is transmitted through the bite of an infected animal [21]. In addition, some viruses are transmitted by vectors, usually blood-sucking insects. For example, DENV Zika (ZIKV) and yellow fever viruses are transmitted to humans through mosquito bites [22,23,24].

After invading the host, viruses navigate to their target tissues and begin invading cells through various mechanisms. A virus’ preference for specific tissues is determined by a combination of factors, including the interaction between the virus’ surface proteins and certain receptors on the host cell. For example, the SARS-CoV-2 virus interacts with the angiotensin converting enzyme 2 (ACE2) receptor through its spike protein [25], and IVA hemagglutinin binds to α-2,6-linked sialic acids on host cells [26,27]. Microenvironmental conditions, such as the pH level, can also influence this process. For example, pH-induced conformational changes in IVA hemagglutinin are critical for the ability of viruses to enter cells [28]. In addition, the activation of inactive viral protein binding after cleavage by tissue-specific secreted proteases may also play an important role in facilitating viral invasion [29]. After attachment, some viruses, such as polio, IVA, and SARS-CoV-2, undergo endocytosis, activating various mechanisms that allow for the release of the viral genome into the cytoplasm of the cell [25,30,31]. Conversely, some enveloped viruses, such as HIV, can fuse their envelope to the host cell membrane and release their genome into the cytoplasm [3]. Within the cell, the viral genome manipulates the host machinery to replicate and generate new viral particles that are released by budding or after host cell lysis, allowing for the infection of new host cells and transmission to new hosts.

### 1.2. Host Signaling and Virus Life Cycles

A virus’ life cycle involves multiple series of events in which it relies on its host’s activities to effectively infect the host cell. During viral infection, a multitude of host components assume pivotal roles in either aiding or impeding the virus’s capacity to successfully carry out its life cycle. Host factors as chemicals, proteins, or cellular machinery that are intrinsic to the host organism have the ability to interact with viral components, thereby influencing the process and outcomes of infection. These factors have the potential to either facilitate viral infection and dissemination or initiate a defensive response to restrict viral infection.

Within the different host factors, signaling molecules draw a lot of attention due to their role in regulating viral life cycles. Host signaling pathways have been acknowledged as therapeutic targets in cancer and viral infections, with tyrosine kinases (TKs) being profoundly explored, and many TK inhibitors (TKIs) have been approved by the FDA for cancer treatment [32,33,34].

To combat viral infections, an integrated strategy that combines immunizations and antiviral drugs is needed. Targeting host enzymes rather than viral targets is appealing due to their genetic stability [35]. As observed with cancer [36], the simultaneous inhibition of several host enzymes or pathways might improve viral resistance. Such a strategy may prompt the development of new technologies to combat current and future viral pandemics. For example, while DENV is resistant to viral protein-targeting drugs, it is susceptible to kinase inhibitors, such as sunitinib and erlotinib [37].

Usually, virus variants are developed due to a lack of proofreading during gene replication or through the recombination of gene segments between co-infecting strains. Although coronavirus possesses some proofreading activity, which may reduce variant occurrence, a number of variants with greater infectivity and immune evading abilities have evolved within months from the primal emergence [38].

Protein kinases play a crucial role in viral infections. Protein phosphorylation is also important in the life cycle of different viruses [39]. Numerous viruses induce or inhibit protein phosphorylation at many different stages of signal transduction pathways from the plasma membrane to the nucleus [40,41]. Phosphorylation might affect protein’s stability, activity, and crucial interactions with other cellular and viral proteins, which play a part in regulating virus infectivity. Viruses such as Epstein-Barr virus (EBV) [42], HCV [42], and SARS-CoV-2 [42] promote mitogen-activated protein kinase (MAPK) phosphorylation. Protein phosphorylation changes have been observed during viral propagation for viral replication. MAPK and ERK-2, for example, phosphorylate the HIV protein p6 at a specific site (Thr 23). Additional viruses seem to activate the MAPK pathway, including DENV [43], coronavirus [44], Venezuelan equine encephalitis virus (VEEV) [45], and enterovirus 71 (EV71) [46], which rely on activated p38 for their replication. Activated p38α interacts with the *N*-terminal region of HCV’s core protein and subsequently phosphorylates it, promoting HCV core protein oligomerization, which is an essential step for viral assembly [47].

Viral infection is associated with an increased production of cytokines, called a cytokine storm. During a cytokine storm, various inflammatory cytokines are produced at much higher levels than normal. This overproduction of cytokines leads to a positive feedback loop on other immune cells, allowing more immune cells to reach the site of injury, which can lead to organ damage. Among the most notable clinical conditions associated with cytokine storms is acute respiratory distress syndrome (ARDS), which is responsible for a significant number of deaths from SARS-CoV-2. Intracellular cell signaling pathways like MAPK p38 mediate the cytokine storm that the virus triggers, which eventually results in immunopathology [48].

MAPK p38 inhibitors exhibited a broad-spectrum antiviral effect against a variety of viruses [47]. In HCV, p38 inhibitors disrupted the MAPK p38α-HCV core protein interaction, efficiently impaired HCV assembly, and prevented normal HCV replication. Similarly, severe fever with the thrombocytopenia syndrome virus (SFTSV), HSV-1, or severe acute respiratory syndrome coronavirus 2 (SARS-CoV-2) also activate p38 MAPK, and p38 inhibitors effectively inhibit their replication [47]. Furthermore, many viruses utilize protein phosphorylation to regulate signaling in order to prevent apoptosis and enhance cellular survival and proliferation [49]. c-Jun *N*-terminal kinase (JNK) signaling influences the replication of some viruses. JNK inhibition, for example, suppresses HSV, HIV, and rotavirus replication [50].

Host receptor tyrosine kinases can serve as entry receptors for some viruses. For example, the tyrosine kinase AXL facilitates the entrance of filoviruses such as EBOV [51]. The epidermal growth factor receptor (EGFR) is a host factor that is frequently required by viruses [52]. Human cytomegalovirus (HCMV) and adeno-associated virus serotype 6 (AAV6) use EGFR as a co-receptor for entry [53,54].

Other receptor tyrosine kinases were implicated in regulating virus replications. The IAV lifecycle is regulated by nerve growth factor receptors (TrkA), human epidermal growth factor receptor 2 (HER2), and platelet-derived growth factor receptor (PDGFR). Tyrphostin AG879, which inhibits TrkA/HER2 signaling, and A9, which targets the PDGFR pathway, were efficient in inhibiting IAV [55]. AP2-associated protein kinase I (AAK1), a member of the numb-associated kinase (NAK) family, and a clathrin-mediated endocytosis regulator are among the other host kinases implicated in the SARS-CoV-2 lifecycle [56]. HCV, DENV, and EBOV infections require the kinases AAK1 and cyclin G-associated kinase (GAK) [57]. Two FDA-approved drugs, sunitinib, and erlotinib, inhibit AAK1 and GAK activity in cultured cells, blocking HCV intracellular trafficking. The combination of sunitinib and erlotinib decreases mortality and morbidity in mice models of DENV and EBOV infection [37]. These findings illustrate the potential of a repurposed, host-targeted approach to combating emerging viruses. Interestingly, the kinase inhibitor baricitinib, which is used to treat rheumatoid arthritis (RA) [58] and inhibits the Janus kinase/signal transducers and activators of transcription (JAK/STAT), additionally blocks NAK and AAK1 kinases [59] and exhibited anti-viral activity in COVID-19 patients [60]. Taken together, multiple host kinases regulate viral life cycles; therefore, inhibitors of key kinases might have antiviral properties.

This review focuses on epigenetic modifications of host proteins caused by viral infection. Moreover, we examined the ability of plant-derived substances to exhibit epigenetic modification activity and consequently their ability to block virus propagation. Plants are a rich source of pharmaceuticals that may be exploited to treat a wide range of human and animal illnesses. Newman et al. (2020) analyze all approved therapeutic drugs for all conditions from 1981 to 2019 and anti-tumor activity from 1946 to 2019. The authors emphasize in their review that a large number of developed drugs, including anti-infective agents, are derived from natural products [61].

As a result, in this review, we investigate the use of plant-derived bioactive compounds that influence epigenetic processes as potential broad-spectrum anti-viral agents.

### 1.3. Epigenetic Modifications

Epigenetics has a key role in cell differentiation, genomic imprinting, and X-chromosome inactivation. In healthy subjects, the epigenetic memory which dictates cell differentiation is acquired in one’s early life and maintained across one’s life; to this aim, a multitude of enzymes, which are regulated by several environmental stimuli (i.e., food intake, chemicals, stress, activators of the immune system, viruses, etc.), are employed. The loss of one’s epigenetic memory can occur during life as it is associated with aging and may lead to susceptibility to diseases [62].

Epigenetics can be both inherited and reversible; it modulates gene expression without any changes in DNA sequences. These modifications can be influenced by environmental factors, such as diet, stress, and exposure to toxins, as well as by changes in gene expression during development. Epigenetic modifications include DNA methylation, histone remodeling, the utilization of alternative histone variants, modifications of histone tails, and the expression of noncoding RNAs, including miRNAs [63]. These activities are together known as epigenetic regulations [64].

#### 1.3.1. DNA Methylation

The most studied epigenetic mechanism is DNA methylation at cytosine residues (5mC); it consists of the addition of a methyl group to the carbon-5 of cytosine in the CpG dinucleotide, catalyzed by DNA methyltransferase enzymes (DNMTs) [65].

DNA methylation is carried out by the 5-cytosine DNMT enzyme using S-adenosylmethionine (SAM) as a methyl donor. In mammals, DNA methylation may occur at cytosines all over the genome [65]. However, in somatic cells, more than 98% of DNA methylation occurs in the vicinity of CpG dinucleotides.

**Figure 1:**
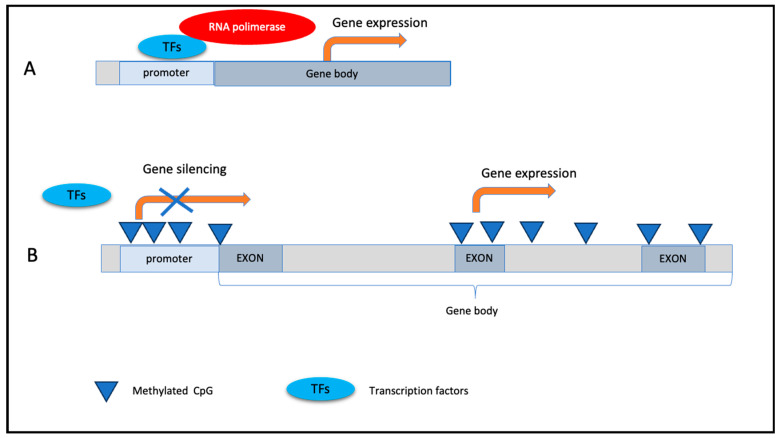
DNA methylation and its modulation of gene expression. Transcription factors recognize gene promoter and bring RNA polymerase to transcript the gene (**A**); DNA methylation at CpG island switch on/off gene according to the locus where methylation occurs (**B**).

DNA methylation has various impacts on gene expression. Generally, when the promoter is non-methylated, the gene expression is switched on (Figure 1A), while if methylation occurs at the promoter level, transcription factors cannot interact at the promoter region and the gene is silenced (Figure 1B). Outside of the promoter sequence, DNA methylation can have different effects on gene expression according to the location of methylated CpGs (Figure 1B). Typically, DNA methylation is removed during the zygote formation and subsequently restored in the embryo at the time of implantation [66]. DNA methylation is essential for proper development and cell differentiation [67,68].

DNA methylation is maintained during replication by DNMT1, while DNMT3a, DNMT3b, and DNMT3c are responsible for de novo methylation and respond to environmental factors. Stress, viruses, bioactive compounds, chemicals, etc., can modulate the activity of DNMTs, leading to a change in the methylome across life. On the other hand, ten-to-eleven translocation (TET) proteins demethylate 5mC to 5-hydroxymethylcytosine (5hmC), leading to physiological and pathological events [69]. These changes in DNA methylation can affect gene expression, thus contributing to the development and progression of diseases and aging. These changes in DNA methylation at specific genes can be used to quantify the biological age of individuals; subjects can exhibit negative age acceleration, leading to a young and healthy biological age or positive age acceleration, which is associated with various diseases [70]. Moreover, defects in DNA methylation are closely associated with cancer. To date, all examined tumor samples studied show a global reduction in DNA methylation [71].

#### 1.3.2. Histone Modifications

**Figure 2:**
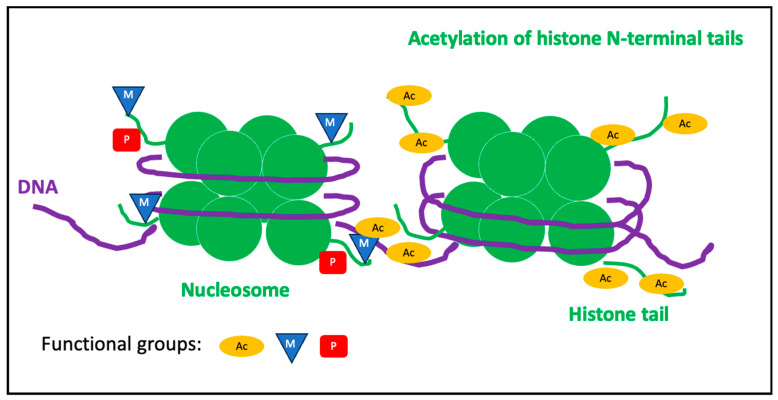
Nucleosome structure and histone modifications. The amino acids chain includes functional groups at specific amino acid residues (histone tails) which modify the interaction between DNA and histones (**left**); acetylation of histone *N*-terminal tails facilitates the transcription initiation (**right**).

Histone modifications are post-translational modifications (PTMs) to histone proteins that include methylation, phosphorylation, acetylation, ubiquitylation, and sumoylation. PTMs on histones result in an altered chromatin structure. The chromatin structure affects the accessibility of the DNA to transcription and replication machineries; histone dynamicity depends on a complex family of enzymes which add functional groups (i.e., acetyl, methyl, phosphate, etc.) to histone tails opening and closing chromatin as well as regulating gene expression. PTMs, such as the addition of acetyl groups to the positive charge of amino acids at the histone tails, mask the electrostatic interaction between DNA and amino acids and relax wrapped DNA to guarantee the accessibility of the complex protein machinery required for gene transcription. Generally, the impact of the other functional groups (i.e., methyl, phosphate, etc.) on the chromatin structure depends on the position and the number of groups (i.e., methyl) bound to amino acid residues at histone tails (Figure 2).

The enzymes involved in histone modifications are histone acetyltransferases (HATs), which relax the chromatin structure, while histone deacetylases (HDACs) remove acetyl groups, leading to chromatin condensation and transcriptional repression. There are five groups of HATs, which have been named after their functions: Guanine nucleotide-binding protein G(t) subunit alpha-1 (GNAT1), MYST, transcription initiation factor TFIID 250 kDa subunit (TAFII250), transcription coactivators CREB binding protein (P300/CBP), and nuclear receptor coactivators like ACTR [72].

An imbalance in histone acetylation is associated with carcinogenesis and the development of other diseases. HDACs, like HATs, play important roles in a variety of cellular processes involving histone H3 and H4. To date, at least four classes of HDACs have been discovered. Class I HDACs include 1, 2, 3, and 8, and class II HDACs include 4, 5, 6, 7, 9, and 10. Sirtuins (SIRTs) are class III enzymes that require NAD+ cofactors and include SIRTs 1–7. The class IV enzyme, which contains only HDAC11, has features of both class I and class II. In cancer cells, the inhibition of HDAC has a significant effect on apoptosis, cell cycle arrest, and differentiation. HDAC inhibitors are being investigated as anticancer agents [73].

Histone methyltransferases (HMTs) can add more than one methyl group to each residue, leading to transcriptional repression or activation; histone demethylases (HDMs) remove methyl groups with a different impact on gene expression. Transcription silencing is also under the control of polycomb group (PcG) proteins, which regulate the chromatin structure; polycomb repressive complex 1 (PCR1) is a histone ubiquitin ligase that modifies histone H2A, while polycomb repressive complex 2 (PCR2) is a histone methyltransferase which transfers methyl groups to specific amino acid residues of histone H3 [74].

Histone tail acetylation at lysine residues has been implicated in increased gene expression in general. However, histone tail methylation activates or represses gene expression depending on which residue is methylated. Examples for such modifications have been found on all four histones (H2A, H2B, H3, and H4). H3 and H4 acetylation are linked to active chromatin, while methylations have numerous functions. For example, histone methylation can affect gene expression.

Histone 3 lysine 9 trimethylation (H3K9me3) and histone 3 lysine 27 trimethylation (H3K27me3) are involved in the repression of gene activity [75]. In contrast, histone 3 lysine 24 trimethylation (H3K4me3) actively marks actively transcribed chromatin [76].

#### 1.3.3. RNA Modifications

RNA transcripts undergo various modifications at the single-nucleotide level, with more than 100 such modifications having been found. These modifications are notably prevalent in transfer RNAs (tRNAs) and other non-coding RNAs (ncRNAs). It has been demonstrated that several of these modifications play a role in regulating the activity of ncRNAs [77]. The range of epitranscriptomic modifications, with a primary focus on methylations, observed on eukaryotic messenger RNAs (mRNAs) is comparatively narrower than that observed on non-coding RNAs (ncRNAs). 

#### 1.3.4. miRNA

**Figure 3:**
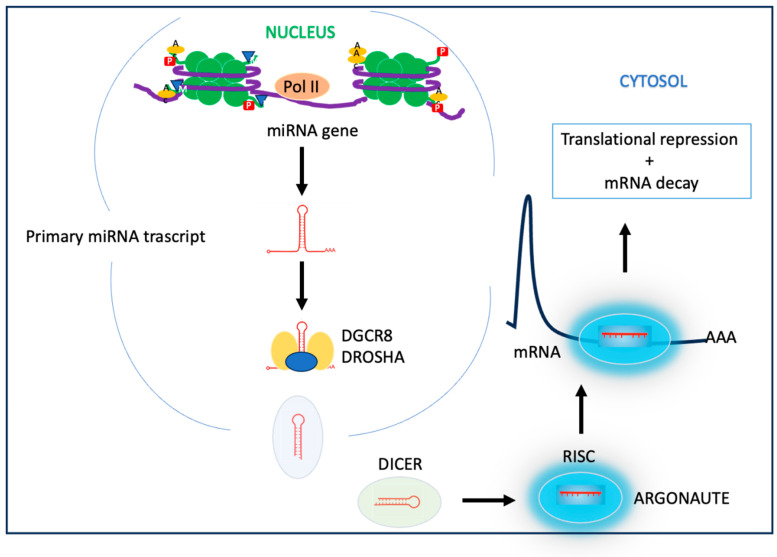
MicroRNA synthesis, translational repression, and degradation of mRNA. MiRNAs are transcribed by RNA polymerase (Pol II), processed by DROSHA and DGCR8 to obtain pre-miRNA; pre-miRNA is transported to the cytosol where DICER enzyme, together with other proteins, processes the miRNA into a single-strand RNA which is incorporated into RISC complex and binds Argonaute. The interaction of miRNA with mRNA is followed by mRNA decay and translational repression.

Non-coding microRNAs (miRNAs) are involved in the post-transcriptional control of gene expression [78]. Several classes of miRNAs have been identified in all cells. MiRNAs, involved in regulating gene expression, are small single-stranded RNAs about 20 ribonucleotides long, possessing the reverse complement of another protein-coding gene’s mRNA transcript. They are transcribed by RNA polymerase II, translocated to the cytosol, then further processed by Dicer and incorporated in the RNA-induced silencing complex (RISC). The miRNA–RISC complex binds the Argonaute protein family and together they interact with 3′-UTR regions of mRNAs; this step leads to translational repression by the degradation of the mRNA [79] (Figure 3). The miRNAs are involved in the regulation of cellular homeostasis and play a role in several diseases due to their ability to silence gene expression.

miRNAs can act as epigenetic modulators by targeting the key enzymes responsible for epigenetic machinery, such as DNMTs, HDACs, and HMTs [80,81,82]. Furthermore, epigenetic machinery, such as DNA methylation, RNA modification, and histone modification, regulate miRNA expression. The miRNA–epigenetic feedback loop is formed by a reciprocal relationship between miRNAs and epigenetic control. Some miRNAs were found to regulate DNMT3a and DNMT3b, as well as methylation-related proteins involved in de novo methylation [83]. For example, miR-148 targets DNMT3b [84]. Members of the miR-29 family were also shown to target DNMT3a and DNMT3b [85].

### 1.4. Viral Infections and Epigenetic Modifications

Research on the human epigenome is becoming increasingly important in cancer, immunology, and infectious diseases [86,87]. Viruses that infect animal cells were shown to cause epigenetic changes, and epigenetic processes influence the majority of virus–cell interactions [88].

After entering the nucleus, viral DNA starts the replication process in close proximity to subnuclear structures known as pro-myelocytic leukemia nuclear bodies (PML-NBs). The PML-NBs serve as aggregation sites for many proteins associated with heterochromatic repression. These proteins facilitate the deposition of repressive heterochromatin on viral DNA, thereby repressing viral transcription. In absence of viral de-repression functions, viral episomal DNA contains repressive chromatin marks and has no active histone marks [89].

Certain DNA viruses, such as HSV and HBV, use viral proteins that disperse or degrade components of PML-NBs to prevent viral gene silencing [90].

When the HSV enters the nucleus, viral DNA exists in a “naked” state, lacking histones. The viral DNA undergoes rapid epigenetic repression, primarily through the action of Daxx-ATRX and HIRA-loaded H3.3 associated with repressive H3K9me3 marks. The inhibition of HSV-1 is neutralized by a two-step process. First, the viral protein VP16 facilitates the recruitment of host proteins, resulting in the formation of a complex that subsequently recruits HDMs, including lysine-specific demethylase 1 (LSD1) and members of the Jumonji domain 2 (JMJD2) family. This recruitment process serves to remove repressive H3K9 marks from viral early promoters. The VP16 complex additionally recruits the methyltransferases Set1 and MLL1, facilitating the activation of H3K4me3 modifications on histone H3 associated with viral DNA. This enables the manifestation of the early viral protein ICP0, which coordinates the subsequent phase of de-repression. The ICP0 protein facilitates the stepwise elimination of the repressive histone modifications H3K9me3 and H3K27me3 throughout the viral DNA genome, enabling successful infection [90]. Recent research therefore supports the notion that PML-NBs play a critical role in the epigenetic suppression of viral DNA.

By inserting their DNA into euchromatic regions of the host cell genome, retroviruses seem to evade epigenetic regulation. This is apparent from the fact that the epigenetic suppression connected to unintegrated retroviral DNA is reminiscent of that found in mutant DNA viruses devoid of necessary defensive mechanisms. Viruses can use epigenetic suppression to enter a regulated, latent state when they infect host cells that are at least momentarily nonpermissive. This can result in the formation of viral reservoirs, which have so far been shown to be unbreakable. It has been suggested that host cells employ the idea of epigenetic repression as an inherent immune response against DNA viruses [91].

In an alternative hypothetical situation, it is possible that viruses use epigenetic repression as an approach to suppress their own gene expression. This could potentially promote the development of latent infections and prevent the production of immunogenic viral proteins [92].

On the other hand, it is well known that epitranscriptomic modifications tend to enhance the functionality of viral messenger RNAs (mRNAs). Consequently, both DNA and RNA viruses have evolved mechanisms to optimize the extent of these modifications on their transcribed genetic material. There are reports of four epitranscriptomic modifications that affect viral gene expression. These modifications include N6-methyladenosine (m6A), 5-methylcytidine (m5C), N4-acetylcytidine (ac4C), and 2′O-methylation of the ribose moiety of all four ribonucleosides (collectively referred to as Nm). Viral RNAs have significantly increased amounts of m6A, m5C, and Nm residues compared with those of cellular mRNAs expressed in infected cells [77,79]. The amount of m5C in viral RNAs was found to be 14–30 times higher compared with that in cellular poly(A)+ RNA. The high prevalence of m6A, m5C, Nm, and possibly ac4C modifications on viral RNAs suggests that viral RNAs have adapted to enhance the incorporation of these epitranscriptomic modifications into their transcripts. This observation strongly implies that these modifications play a crucial role in facilitating various stages of the viral replication cycle [93].

HDACs that remove acetyl groups from histone proteins and alter gene expression result in interferences with viral replication. Thus, HDAC inhibitors have been shown to be promising antiviral drugs. While there are several synthetic HDAC inhibitors on the market, there are also natural bioactive compounds that have HDAC inhibitory action and, consequently, anti-viral activity.

**Figure 4:**
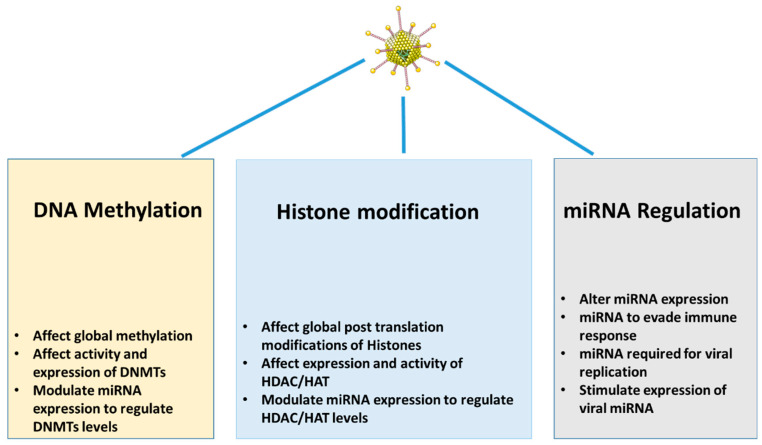
Viral infection and epigenetic machinery. Viral infection affects global DNA methylation and the expression and activity of DNMTs. In addition, viral infection affects global histone modifications and modulates the expression and activity of HAT and HDAC. Viral infection also modulates the expression of miRNA, which is required to evade immune response and to promote viral replication.

Of note, SARS-CoV-2 infection raised the levels of the methylated histones H3K9me3 and H3K27me3 in A549 cells [94]. ORF8 encodes the SARS-CoV-2 protein, which acts as a histone mimic of the ARKS motifs in histone H3 to impair host cell epigenetic control. ORF8, linked with chromatin, disturbs histone post-translational modification regulation and increases chromatin compaction. Interestingly, SARS-CoV-2 without ORF8 is linked to a lower severity of COVID-19 [94] (Figure 4).

### 1.5. Virus Infection Promotes Epigenetic Modifications in the Immune System and Host Genes

To survive and replicate in their host, viruses must use mechanisms to bypass the immune system. Some virus families engage the immune system through a variety of epigenetic mechanisms. The H3N2 IAV, for example, demonstrated the ability to block the host’s innate immune response by interfering with gene expression. Other cancer-causing animal viruses frequently impair cellular epigenetic mechanisms by reducing tumor suppressor genes and activating viral or host cell oncogenes.

Interferons (IFNs) serve as crucial anti-viral mediators as they stimulate interferon-stimulated genes (ISGs) and consequently trigger pathogen-driven immune responses [95,96]. During infection, viruses develop antagonistic mechanisms that neutralize certain ISG effectors [97]. IFN and innate immune responses are regulated by epigenetic modifications and chromatin remodeling complexes. H3K27me3 levels increased in MERS-CoV-infected cells while H3K4me levels decreased, indicating the antagonistic mechanisms targeting the IFN innate immune response [98]. Differential histone mark occupancy at ISG promoters indicated that promoter regions had additional H3K4me monomethylation than repressive H3K27me3 marks, favoring accessible chromatin and promoting active transcription and ISG expression [99]. As DNA methylation and histone modifications govern ACE2 expression, DNMT1, HAT1, HDAC2, and KDM5B are possible viral targets for modulating the host immune response [100].

Virus-mediated epigenetic modifications have additionally been linked to cancer induction. The HBV is linked with HCC together with abnormal DNA methylation in the host genome. HBV-infected cells and tumors exhibit greater levels of DNMT1, DNMT3a, and DNMT3b. HBV X antigen (HBXAg) has been reported to induce DNMT1 and DNMT3a expression. HBXAg has demonstrated effective transcription repression from CpG-methylated E-cadherin and p16INK4A gene regulatory elements.

Human papillomavirus (HPV) strains HPV16 and 18 have been linked to the development of cervical carcinoma. A keratinocyte cell line with HPV16 episomes has been shown to have a lower level of E-cadherin [101]. This downregulation requires the oncoprotein HPV-E7, which was reversible following treatment with the DNMT inhibitor 5-aza-deoxycytidine [101]. Furthermore, E7 has been demonstrated to alter DNMT1 levels [101], bind directly to DNMT1, and participate in DNA methyltransferase activity [102]. E7 also stimulates the expression of the H3K27 methyltransferase EZH2 [103]. Interestingly, the gene p16INK4A, which is typically suppressed by EZH2 via H3K27me3 in cycling cells and is commonly hyper-methylated in cancer, is frequently overexpressed in HPV-positive carcinoma [104] (Figure 4).

Simian vacuolating virus 40 (SV40) is a polyomavirus that infects both monkeys and humans. It is also suspected to induce epigenetic modifications in host cells. SV40, like HPV, is linked to RASSF1 promoter DNA methylation, with the presence of SV40 large T-antigen (Tag) sequences correlating with high levels of DNA methylation [105]. Furthermore, the expression of large T antigens upregulates DNMT1, promoting DNA methyltransferase activity and genomic methylation [106] (Figure 4).

Adenoviruses undergo a lytic rather than a dormant life cycle, and the viral oncoprotein small E1A was shown to reduce histone 3 lysine 18 acetylation (H3K18Ac) and block the transcription of multiple genes [107].

Interestingly, the FDA authorized a variety of epigenetic modifiers as DNMT inhibitors (azacytidine, decitabine) and HDAC inhibitors (vorinostat, romidepsin, belinostat, panobinostat) [108]. Decitabine and 5-aza-2-deoxycytidine (5-azadC) are nucleoside-based DNMT inhibitors widely used for reducing inflammation and IFN response in macrophages [109]. Notably, decitabine has recently been incorporated in a clinical study for the treatment of COVID-19 pneumonia and ARDS (CTI: NCT04482621).

### 1.6. Viral Infection Affects Host miRNA Expression

In response to viral infection, the expression levels of host miRNAs vary. These up/downregulated miRNAs directly or indirectly target the viral genome to regulate viral replication and to modulate the innate immune system during viral infection [110].

A recent discovery highlights the critical function of miRNAs in viral infection and disease progression. Consequently, numerous researchers have been engaged in investigating the potential utilization of these microRNAs (miRNAs) as diagnostic or therapeutic instruments. Modulating the expression of microRNAs (miRNAs) in the context of anti-viral therapy has proven to be a challenging endeavor due to the inherent variability exhibited by these molecules under diverse circumstances. A recent study presented evidence supporting the possibility of using eight distinct miRNAs (miR-122, miR-155, miR-223, miR-150, miR-199, miR-149, miR-29, and miR-let7) to serve as biomarkers for HIV, HCV, or HIV/HCV co-infection [111]. Some of above-mentioned miRNAs, such as members of the miR-29 family, are targeting DNMT3a and DNMT3b [85] (Figure 4).

The most prevalent miRNA in the liver is miR-122 [112], which accounts for 60–70% of the total miRNA in hepatocytes. Numerous studies have revealed that miR-122 is needed for HCV and HIV replication in infected cells [113,114,115].

The MiR-223 expression is downregulated during IAV and HBV infection, as well as in inflammatory bowel disease, type 2 diabetes, leukemia, and lymphoma [116]. A growing body of data shows that miR-223 has a role in controlling inflammation and preventing collateral damage during infection. Granzyme B, IKK, and STAT3 [116] are all validated targets for miR-223 that have impacts on inflammation and infection. miRNA-223 can also target viruses directly, such as HIV [115]. Recently, miR-223 was shown to be increased in HIV/HCV co-infection, although its significance in co-infection remains unknown [115]. Furthermore, during SARS-CoV infection, miRNA-223-3p was identified as a host miRNA involved in the control of the lung inflammatory response mediated by the envelope (E) protein [117]. Inhibiting miRNA-223 in infected animals using antisense RNAs resulted in alterations in the expression of host components implicated in inflammation (cytokines, chemokines, the nucleotide-binding domain, leucine-rich-containing family, and pyrin domain containing 3 (NLRP3) inflammasome) as well as the resolution of pulmonary edema ion transporter cystic fibrosis transmembrane conductance regulator (CFTR). These findings demonstrate the role of miRNA-223 in the control of SARS-CoV-induced pathogenic processes and support the therapeutic potential of miRNA inhibition [117]. Interestingly, most viruses produce their own ncRNAs, which bind with proteins essential for their activity and stability. These ncRNAs control viral replication, persistence, host immune evasion, and cellular transformation [118]. Herpesvirus saimiri encodes HSUR 1, an uracil-rich ncRNA with a sequence complementarity to miR-27. Binding to HSUR 1 triggers miR-27 cleavage, activating T cells during HSV infection [119,120,121].

## 2. Plant-Derived Anti-Viral Activity

Although vaccines are used to combat certain viruses, antiviral therapy is still a common approach [122]. Interest in herbal products with antiviral activity has increased significantly, and in recent years, more than 50% of approved antiviral drugs have been produced from herbal sources [123,124]. Indeed, they are particularly rich in compounds with a great structural diversity.

Plant-derived compounds offer a wide range of chemical structures and bioactive molecules, increasing the likelihood of discovering novel antiviral compounds. This approach is consistent with the increasing importance of sustainable and environmentally friendly drug discovery and development. Plant-derived compounds can exhibit a broad spectrum of activity, reduce drug resistance, and provide a multi-target approach that increases the efficacy of antiviral therapies. Combining these therapies with conventional antiviral drugs could lead to synergistic effects and improved treatment outcomes. However, extensive preclinical and clinical studies are still needed to determine the safety and efficacy of plant-based epigenetic modulators as antiviral therapies. Plant-derived products are mainly essential oils (EOs), extracts, and isolated compounds that have been extensively studied for their antiviral properties against different viral strains [125,126].

EOs and extracts from different plant families such as Lamiaceae, Myrtaceae, Asteraceae, Brassicaceae, Rutaceae, Apiaceae, and Geraniaceae were found to be highly effective (Table 1). Among them, the essential oils of *Illicium verum* Hooker f. and *Rosmarinus officinalis* L. were extremely effective against HSV-1 with IC_50_ values of 1 and 0.18 µg/mL, respectively [127,128]. In addition, *Cymbopogon citratus* (DC.) Stapf, *Cananga odorata* (Lam.) Hook.f. & Thomson, and *C. nardus* (L.) Rendle showed great efficacy against HIV with IC_50_ values of 0.61, 0.60, and 1.2 µg/mL, respectively [129,130]. Finally, *Eucalyptus globulus* Labill. proved to be highly effective against Coxsackie virus B3 with an IC_50_ of 0.7 µg/mL.

As for the plant extracts listed in Table 2, most of them showed strong antiviral activity. For example, extracts of *Schinus terebinthifolia* Raddi and *Quercus persica* Jaub. & Spach inhibited HSV-1 with an IC_50_ of 0.21 and 0.26 µg/mL, respectively [129].

Comparing the data reported in Table 1 and Table 2, herbal EOs and extracts are more effective than pure isolated compounds in some cases. EOs are usually complex mixtures of volatile secondary metabolites such as terpenes, alcohols, ethers, aldehydes, ketones, or esters. The extracts are also complex mixtures of organic compounds that are extracted depending on the polarity and solvent used. Consequently, the activity of EOs and extracts is not due to a single component but may be associated with the synergistic action of two or more components with antiviral activity.

Herbal products exert their antiviral activity with different modes of action. They may inhibit virus attachment, penetration, and entry into the host cell or inhibit other intercellular cell signaling pathways [131]. Other modes of action include the disruption of the viral life cycle or inhibition of other essential enzymes for viral reproduction [132].

A wide range of compounds are found in plants and their extracts, some of which have been isolated and tested to identify the main contributors to antiviral activity. Most of them belong to the classes of polyphenols, terpenes, or alkaloids. In some cases, the compounds have shown strong potential against several viruses.

Among the terpenes, raoulic acid purified from *Raoulia australis* Hook. F. purified extract showed antiviral activity against five virus strains, i.e., HRV2, HRV3, CB3, CB4, and EV71 (IC_50_ values of <0.1, 0.19, 0.33, 0.40, and <0.1 µg/mL, respectively) [133]. In addition, farnesol, β-eudesmol, and carvacrol showed great potency against HSV-1 with IC_50_ values of 0.25, 3.5, and 6 μg/mL, respectively [127]. The classes of polyphenols and flavonoids contain the most compounds with antiviral activity. For example, chebulagic acid, a tannin derived from *Terminalia chebula* Retz, was effective against viruses that use glycosaminoglycans for entry such as HCMV, HCV, DENV-2, and RSV, showing IC_50_ values of 25.50, 12.16, 13.11, and 0.38 µM, respectively [134]. Baicalein, belonging to the flavonoid class, potently suppressed HCMV and HSV1, and the IC_50_ were determined to be 2.2 and 5 µM, respectively [135,136].

Finally, alkaloids, which are small nitrogen-containing molecules, have a molecular basis of specificity and the ability to act on multiple viruses with different mechanisms of action [123]. Among them, tetrandrine and cepharanthine showed strong potential against HcOV (IC_50_ values of 0.33 μM and 0.83 μM, respectively), while berberine was effective against HCMV, HSV1, and HSV2 (IC_50_ values of 2.65, 6.77, and 5.04 μM, respectively) [137,138,139].

**Table 1:** Plant-derived essential oils (EOs) with antiviral activity.

Plant	Plant Part	Main Bioactive Constituents	Virus	IC_50_^a^	SI ^b^	Mode of Action	References
*Aloysia citriodora* Palau (Verbenaceae)	nr	geranial (18.9%), neral (15.6%), limonene (10.7%), 1,8-cineole (5.0%), spathulenol (4.7%), geraniol (2.7%)	Yellow fever virus	19.4 μg/mL	2.6	nr ^c^	[140]
*Artemisia kermanensis* Podlech (Compositae)	aerial parts	α-thujone (13.8%), camphore (10.2%), β-thujone (6.2%), p-Mentha-1,5-dien-8-ol (4.4%)	HSV-1	0.004%	66.4	nr	[128]
*Ayapana triplinervis* (Vahl) R.M.King & H.Rob. (Compositae)	aerial parts	thymohydroquinone dimethyl ether (87.1%), α-phellandrene (2.0%), β-selinene (1.9%)	ZIKV	38 μg/mL	12.5	inhibitor of internalization process	[141]
*Cananga odorata* (Lam.) Hook.f. & Thomson (Annonaceae)	nr	benzyl salicylate (49.3%), benzyl benzoate (18.7%), linalool (16.6%), α-gurjunene (7.1%)	HIV-1	0.60 µg/mL	nr	nr	[129]
*Cinnamomum zeylanicum* Blume (Lauraceae)	leaves	eugenol (nr)	H1N1	<3.1 µL/mL	>4	intercellular	[142]
*Citrus × bergamia* Risso & Poit. (Rutaceae)	fruit peel	(–)-linalyl acetate (nr), (–)-linalool (nr), (+)-limonene (nr), γ-terpinene (nr), β-pinene (nr), α-pinene (nr), α-terpinene (nr)	H1N1	<3.1 µL/mL	>5	intercellular	[142]
*Cymbopogon citratus* (DC.) Stapf (Poaceae)	nr	*cis*-citral (59.2%), β-pinene (22.5%), cis-verbenol (6.1%), nerol (5.0%)	HIV-1	0.61 µg/mL	1.1	nr	[129]
*Cymbopogon flexuosus* (Nees ex Steud.) W.Watson (Poaceae)	grass	geranial (nr), neral (nr)	H1N1	<3.1 µL/mL	>4	nr	[142]
*Cymbopogon nardus* (L.) Rendle (Poaceae)	nr	nr	HIV-1	1.2 mg/mL	nr	nr	[130]
*Dysphania ambrosioides* (L.) Mosyakin & Clemants (Amaranthaceae)	aerial parts	cis-ascaridole (60.3%), *m*-cymene (22.2%), α-terpinene (1.8%), thymol (1.1%)	Coxsackie virus B4	21.7 μg/mL	74.3	nr	[143]
*Eucalyptus caesia* Benth. (Myrtaceae)	aerial parts	1,8-cineole (40.2%), *p*-cymene (14.1%), γ-terpinene (12.4%), α-pinene (7.7%), terpinen-4-ol (5.6%)	HSV-1	0.007%	38.8	nr	[128]
*Eucalyptus globulus* Labill. (Myrtaceae)	leaves	1,8-cineole (nr), α-pinene (nr)	H1N1	<50 µL/mL	>0.5	intercellular	[129]
		1,8-cineole (68.0%), globulol (5.4%), trans-pinocarveol (4.6%), α-pinene (3.7%)	Coxsackie virus B3	0.7 mg/mL	22.8	intercellular	[144]
*Fortunella margarita* Lour. Swingle (Rutaceae)	fruits	terpineol (55.5%), *τ*-carveol (5.5%), limonene (1.7%), muurolene (5.5%), cadinene (2.0%)	H5N1	6.8 µg/mL	nr	nr	[145]
*Illicium verum* Hooker f. (Schisandraceae)	fruits	(*E*)-anethole (80.0%)	HSV-1	1 µg/mL	160	intercellular	[127]
*Lallemantia royleana* (Benth.) Benth. (Lamiaceae)	aerial parts	(*E*)-pinocarvyl acetate (26.0%), pinocarvone (20.0%), verbenone (7.1%), (*E*)-β-ocimene (4.1%), (*E*)-carveol (5.3%), 3-thujen-2-one (5.1%), pulegone (4.4%), (*Z*)-carveol (3.5%), linalool (3.4%)	HSV-1	0.011%	6.4	intercellular	[146]
*Lavandula officinalis* Chaix (Lamiaceae)	flowers	linalyl acetate (nr), linalool (nr)	H1N1	<3.1 µL/mL	>8	intercellular	[129]
*Lippia alba* (Mill.) N.E.Br. ex Britton & P.Wilson (Verbenaceae)	nr	carvone (39.7%), limonene (30.6%), bicyclosesquiphellandrene (8.9%), piperitenone (4.5%), piperitone (2.8%), β-bourbonene (1.7%)	Yellow fever virus	4.3 μg/mL	30.6	intercellular and intracellular	[140]
*Lippia graveolens* Kunth (Verbenaceae)	nr	carvacrol (56.8%), o-cymene (32.2%), γ-terpinene (3.7%)	HSV-1	99.6 µg/mL	7.4	intercellular	[147]
			ACVR-HHV-1	55.9 µg/mL	13.1	intercellular	
			Bovine viral diarrhoea virus	78 μg/mL	7.2	intracellular	
			Respiratory syncytial virus	68 μg/mL	10.8	intercellular	
			Bovine herpes virus 2	58.4 μg/mL	9.7	intercellular and intracellular	
*Melaleuca alternifolia* (Maiden & Betche) Cheel (Myrtaceae)	leaves	nr	HSV-1	13.2 µg/mL	43	intracellular	[148]
*Mentha suaveolens* Ehrh. (Lamiaceae)	leaves	piperitenone oxide (86.8%), α-cubebene (2.1%), pulegone (1.4%), limonene (1.4%), caryophyllene (1.3%)	HSV-1	5.1 µg/mL	67	intracellular	[149]
*Osmunda regalis* L. (Osmundaceae)	aerial parts	hexahydrofarnesyl acetone (11.8%), 2,4-di-t-butylphenol (6.8%), phytol (6.5%), neophytadiene (4.6%), 1-octadecene (4.4%), 1-eicosene (4.4%), 1-hexadecene (4.1%)	Coxsackie virus B4	2.2 μg/mL	789.8	nr	[150]
*Pelargonium graveolens* L’Hér. (Geraniaceae)	flowering aerial parts	citronellol (nr), geraniol (nr)	H1N1	<3.1 µL/mL	>21	intercellular	[129]
*Pulicaria vulgaris* Gaertn. (Compositae)	aerial parts	thymol (50.2%), p-menth-6-en-2-one (carvotanacetone, 20.2%), thymol isobutyrate (16.9%), menthan-2-one (4.3%)	HSV-1	0.001%	1	intercellular	[146]
*Rosmarinus officinalis* L. (Lamiaceae)	aerial parts	α-pinene (23.9%), verbenon (15.4%), camphor (11.0%), p-cymene (7.5%), 3-octanone (5.6%)	HSV-1	0.006%	46.1	nr	[128]
		eucaliptol (50.6%), camphor (13.3%), α-pinene (10.1%), β-pinene (7.7%), camphene (4.6%)	HIV-1	0.18 µg/mL	3.6	nr	[129]
*Salvia desoleana* Atzei & V.Picci (Lamiaceae)	aerial parts	linalyl acetate (30.2%), germacrene D (18.7%), α-terpinyl acetate (16.8%), 1,8-cineole (10.2%), linalool (5.1%)	Acyclovir-resistant HSV-2	28.6 µg/mL	55.2	intercellular and intracellular	[151]
*Satureja hortensis* L. (Lamiaceae)	aerial parts	carvacrol (32.4%), γ-terpinene (32.0%), thymol (10.0%), p-cymene (6.6%), α-pinene (4.3%)	HSV-1	0.008%	32.2	nr	[128]
*Sinapis arvensis* L. (Brassicaceae)	flowers	cubenol (14.3%), 2-phyenyl isothiocyanate (7.5), dimethyl trisulfide (5.2%), thymol (4.6%), δ-cadinene (3.4%)	HSV-1	0.035%	1.5	intercellular	[146]
*Thymbra capitata* (L.) Cav. (Lamiaceae)	aerial parts	cinnamaldehyde (nr), carvacrol (nr)	HSV-1	17.6 µg/mL	6.0	intercellular	[152]
			HSV-2	18.6 µg/mL	6.9	intercellular	
*Thymus vulgaris* L. (Lamiaceae)	aerial parts	1,8-cineole (nr), terpenyl acetate (nr), borneol (nr)	H1N1	<3.1 µL/mL	>4	intercellular	[142]
		thymol (37.8%), iso-thymol (23.2%), γ-terpinene (13.2%), β-caryophyllene (4.2%), linalool (3.3%)	HIV-1	1.30	1.6	nr	[129]
*Zataria multiflora* Boiss. (Lamiaceae)	aerial parts	thymol (33.1%), carvacrol (25.9%), p-cymene (11.3%), α-pinene (3.9%)	HSV-1	0.003%	55.4	nr	[128]

^a^ IC_50_, half maximal inhibitory concentration; ^b^ SI, selectivity index; ^c^ nr, not reported.

**Table 2:** Plant-derived extracts with antiviral activity.

Plant	Plant Part	Extract	Main Bioactive Constituents	Virus	IC_50_^a^	SI ^b^	Mode of Action	References
*Agrimonia pilosa* Ledeb. (Rosaceae)	whole plant	ethanol extract	nr ^c^	H1N1, HV A-B	0.5–1 μg/mL	nr	block uncoating process	[153]
*Aloe vera* (L.) Burm.f. (Xanthorrhoeaceae)	leaf	Gel	nr	HSV-1	5%	nr	replication inhibitor	[154]
*Arachis hypogaea* L. (Leguminosae)	peanut skin	ethanol extract	nr	H1N1	1.3 μg/mL	5.2	early stages of infection inhib.	[155]
*Avicennia marina* (Forssk.) Vierh. (Acanthaceae)	leaf	methanol extract	nr	HSV-1	9 μg/mL	9.1	viral replication inhib.	[156]
				HIV	15 μg/mL	5.2	interference with replication cycle	
*Centella asiatica* (L.) Urb. (Apiaceae)	leaf	water extract	nr	HIV	36 μg/mL	nr	immunomodulatory effect	[157]
		alcoholic extract		HIV	8 μg/mL			
*Combretum adenogonium* Steud. ex A.Rich. (Combretaceae)	root	water/ethanol extract	nr	HIV-1	24.7 μg/mL	nr	protease inhibitor	[158]
*Copaifera reticulate* Ducke (Leguminosae)	stem, bark and leaf	water/ethanol extract	phenolics, alcohols, organic acids	HSV-2	50 μg/mL	nr	block virus attachment	[159]
*Cornus canadensis* L. (Cornaceae)	leaf	water/ethanol extract	tellimagrandin I and other hydrolysable tannins	HSV-1	9 μg/mL	nr	virus absorption inhibitor	[160]
*Embelia ribes* Burm.f. (Primulaceae)	fruit	ethylacetate extract	embelin	H1N1	0.2 μg/mL	32	block virus entry	[161]
*Epimedium koreanum* Nakai (Berberidaceae)	bark	water extract	nr	HSV	0.20 μg/mL	23.5	immunomodulatory effect	[162]
*Equisetum giganteum* L. (Equisetaceae)	root and stem	water/ethanol extract	phenolics, alcohols, organic acids	HSV-2	18 μg/mL	nr	block virus attachment	[159]
*Eupatorium perfoliatum* L. (Compositae)	aerial part	hydroalcoholic extract	nr	H1N1	7 μg/mL	52	viral attachment inhibitor	[163]
*Euphorbia hirta* L. (Euphorbiaceae)	aerial part	methanol extract	nr	HIV-1	38 μg/mL	nr	RT inhib.	[164]
				HIV-2	22 μg/mL			
*Ficus religiosa* L. (Moraceae)	bark	methanol extract	nr	HSV-2	5.2 μg/mL	31.1	nr	[165]
*Hemidesmus indicus* L. Br. ex Schult. (Apocynaceae)	root	water/methanol extract	2-hydroxy-4-methoxybenzaldehyde (0.41 mg/g), 3-hydroxy-4- methoxybenzaldehyde (0.16 mg/g)	HSV-1	66.8 μg/mL	nr	anti-ER α-glucosidase inhib.	[166]
			2-hydroxy-4-methoxybenzaldehyde (0.41 mg/g), 3-hydroxy-4- methoxybenzaldehyde (0.16 mg/g)	HSV-2	70.6 μg/mL			
*Jatropha multifida* L. (Euphorbiaceae)	stem	water extract	nr	H1N1	25 μg/mL	nr	block virus entry	[167]
*Paeonia lactiflora* Pall. (Paeoniaceae)	root	ethanol extract	nr	H1N1	0.016 mg/mL	13.5	block several stages of infection	[168]
*Pedilanthus tithymaloides* (L.) Poit. (Euphorbiaceae)	leaf	methanol extract	2-(3,4-dihydroxy-phenyl)-5,7-dihydroxy-chromen-4-one or luteolin	HSV-2	48.5 μg/mL	9.0	NF-κB signalling pathway modulation	[169]
*Prunella vulgaris* L. (Lamiaceae)	flowers	water extract	nr	HIV	0.8 μg/mL	nr	early post-virus binding interference	[170]
*Prunus dulcis* (Mill.) DA Webb (Rosaceae)	almond skin	methanol/HCl extract	nr	HSV-1	0.04 mg/mL	nr	block virus entry	[171]
*Quercus brantii* Lindl. (Fagaceae)	fruit	chloroform extract	nr	HSV-1	2.9 μg/mL	nr	block virus entry	[172]
*Quercus persica* Jaub. & Spach (Fagaceae)	fruit	water/ethanol extract	nr	HSV-1	0.26 μg/mL	nr	attachment inhib.	[162]
*Rhus aromatica* Aiton (Anacardiaceae)	root/stem bark	water extract	gallic acid	HSV-1	0.0005%	nr	nr	[173]
*Rhus aromatica* Aiton (Anacardiaceae)	root/stem bark	water extract	gallic acid	HSV-2	0.0043%	nr	nr	[173]
*Schinus terebinthifolia* Raddi (Anacardiaceae)	bark	water/ethanol extract	condensed tannins (catechin, 5.4 mg/L)	HSV-1	0.21 μg/mL	<49.0	virucidal effect	[174]
*Solanum melongena* L. (Solenaceae)	peel	ethanol/HCl extract	delphinidin-3-rutinoside (90.3–115.0 μg/mg), chlorogenic acid (24.5–60.7 μg/mg)	HSV-1	83.4 μg/mL	nr	reduction of viral protein expression	[175]
*Strychnos pseudoquina* A. St.-Hil. (Loganiaceae)	stem bark	ethyl acetate extract	nr	HSV-1	5.29 μg/mL	nr	interference with various step of virus cycle	[176]
*Strychnos pseudoquina* A. St.-Hil. (Loganiaceae)	stem bark	ethyl acetate extract	nr	HSV-2	6.55 μg/mL	nr	interference with various step of virus cycle	[176]
*Tanacetum parthenium* (L.) Sch.Bip. (Compositae)	aerial parts	water/ethanol extract	chlorogenic acid, flavonoids (aglycones and glycosylated flavonoids), parthenolide	HSV-1	3.1 μg/mL	nr	viral replication inhib.	[177]
*Taxodium distichum* (L.) Rich. (Cupressaceae)	cone, leaf, stem	water extract	nr	H1N1	0.05 mg/mL	5.6	block virus entry	[178]
*Vachellia nilotica* (L.) P.J.H. Hurter & Mabb. (Leguminosae)	bark	methanol extract	nr	HSV-2	4.71 μg/mL	30.6	block virus attachment	[179]
*Vachellia nilotica* (L.) P.J.H. Hurter & Mabb. (Leguminosae)	bark	methanol extract	nr	HPV-16	1.80 μg/mL	32.6	block virus attachment	[179]
*Vachellia nilotica* (L.) P.J.H. Hurter & Mabb. (Leguminosae)	bark	methanol extract	nr	HSV-2 acyclovir resistant	6.71 µg/mL	21.5	block virus attachment	[179]
*Vigna radiata* (L.) R.Wilczek (Leguminosae)	sprout	methanol/HCl extract	nr	HSV-1	7.62 μg/mL	nr	virucidal effect	[180]

^a^ IC_50_, half maximal inhibitory concentration; ^b^ SI, selectivity index; ^c^ nr, not reported.

This review focuses on plant-derived substances exhibiting anti-viral activity by targeting host functions and in particular, the epigenome machinery.

### 2.1. Andrographolide 

Andrographolide (AGL), has been used in traditional medicine for millennia to cure a range of diseases, such as colds, flu, and malaria [181,182]. AGL contains many substances that have an antiviral effect [181,182]. AGL inhibited HIV, HSV-1, HBV, HCV, ZIKV, Chikungunya virus (CHIKV), and IAV [183]. AGL and its analogues reduce ZIKV and DENV infections and have been linked to a decrease in HSPA1A expression and an increase in PGK1 protein expression [184]. Foot-and-mouth disease (FMD) is caused by the FMD virus (FMDV) and has a detrimental impact on livestock all over the world. FMDV was suppressed by AGL in BHK-21 cells. AGL reduced FMDV 3Cpro activity as monitored in an intracellular protease assay. Furthermore, AGL greatly inhibited the 3Cpro’s interferon (IFN) antagonistic effect by inhibiting the expression of interferon-stimulating genes (ISGs) [185]. AGL prevents EBV reactivation in EBV-positive cancer cells by suppressing EBV lytic genes, most likely via histone modifications such as H3-K9 modification and H3-K27 methylation [186]. AGL has been shown to prevent infections of DENV [184], ZIKV, and other arboviruses [187]. AGL and its derivative showed significant activity against IAV including the H5N1 avian IVA both in vitro and in vivo [188]. Moreover, IVA-induced inflammation was inhibited by AGL in a murine model through the NF-κB and JAK-STAT signaling pathways [189].

AP has shown promising results in treating liver diseases, including viral hepatitis, liver injury, liver fibrosis, fatty liver, and liver cancer. However, clinical applications of AP are rare due to its poor solubility and low bioavailability.

AP activity mediated the modulation of miRNA. Specifically, MiR-377, which controls heme oxygenase-1 (HO-1), was significantly decreased by AP-induced nuclear factor erythroid 2-related factor 2 (Nrf2) activity.

In addition, AP downregulates miR-433 which modulates the glutathione cysteine ligase. On the other hand, AP upregulates miR-17 and miR-224 which regulate the expression of thioredoxin. Moreover, AP upregulates miR-181a which regulates glutathione peroxidase [190].

### 2.2. Apigenin

Apigenin (4′,5,7-trihydroxyflavone) is a flavone found in a variety of plants, including medicinal plants [191]. Apigenin displayed a potent HDAC inhibitor activity in human prostate cancer PC-3 cells, specifically decreasing HDAC1 and HDAC3 activity [192]. It also increased the global acetylation of histones H3 and H4 and directed histone H3 hyperacetylation to the p21/WAF1 promoter [192]. Furthermore, molecular studies revealed that apigenin enhances acetylated H3, particularly in the p21WAF1/CIP1 promoter region, leading to upregulating p21WAF1/CIP1 transcription [193]. Moreover, apigenin inhibited the expression of miR-155, one of the miRNAs induced by virus infections, which resulted in an increase in SHIP-1 expression and thus impacted anti-tumor immune responses in the bone marrow (BM) and tumor microenvironment (TME) [194].

Apigenin exhibited antiviral properties against IAV, human rhinovirus (HRV), HSV, enterovirus, HBV, HCV, EV71, and SARS-CoV-2 [195,196,197,198]. Its anti-viral activity is attributed, in part, to the inhibition of HDAC activity and chromatin remodeling [192,199]. Apigenin, linalool, and ursolic acid showed a strong anti-viral activity towards coxsackievirus B1 (CVB1) [200]. Apigenin reduced the expression of miRNAs like miR-103, whose overexpression is linked to glucose intolerance, by hindering miR-103’s maturation and preventing ERK from phosphorylating the TRBP (trans-activating response RNA-binding protein) [201].

In addition, apigenin also reduces miR-122 levels in vitro [202]. Because miR-122 overexpression is essential for HCV and HIV replication [113,114,115], apigenin may have anti-HIV and -HCV efficacy by downregulating miR-122.

### 2.3. Baicalein

Baicalein is a flavonoid derived from the roots of *Scutellaria baicalensis* Georgi., a traditional Chinese medicinal herb [203]. It has been investigated for its anti-viral properties against a variety of viruses, including HBV [204], HIV [205], DENV [206], and HSV-1 [207]. Baicalein was reported to inhibit DNMT and HDAC and thereby influence epigenetic modifications [208,209]. Baicalein inhibited HDAC-1/8, causing growth suppression and differentiation induction in AML cell lines. Baicalein might activate HDAC-1 degradation mediated by the ubiquitin-proteasome pathway, thereby increasing histone H3 acetylation [208]. Additionally, baicalein regulates HDAC activity by upregulating the levels of miR-3,178 [210]. Later, an experimental analysis revealed that HDAC10 is a target gene of miR-3,178 [209]. Furthermore, when exposed to high glucose concentrations, baicalin controlled the N6-adenosine-methyltransferase (METTL3)/hexokinase domain containing 1 (HKDC1)/JAK2/STAT1/caspase-3 pathway in liver cancer cells [209]. Baicalein exerts a potent antiviral activity against DENV. It exhibits activity against DENV adsorption and the intracellular replication of DENV-2 [206].

### 2.4. Berberine

Berberine (BBR) is a quaternary ammonium salt of the protoberberine group of benzylisoquinoline alkaloids found in plants such as *Berberis vulgaris* L. [211], which exhibited anticancer activity by affecting epigenetic regulation and AMPK activation [212]. Berberine has exceptional anticancer effects via affecting the enzyme involved in histone acetylation and methylation in acute myeloid leukemia (AML) cell lines [213] and the suppression of SIRT1 deacetylases in a p53-dependent manner [214]. Berberine inhibited miR-21 expression and promoted integrin β4 (ITGβ4) and programmed cell death 4 (PDCD4) protein expression in colon cancer cell lines. The overexpression of miR-21 reduced the anti-cancer effects of berberine on cancer cells [215].

BBR was reported to influence multiple biological activities, including anticancer, anti-inflammatory, and anti-viral activities [216]. BBR targets multiple steps of the viral life cycle, rendering it an excellent candidate for use in innovative anti-viral drugs and therapies. BBR was discovered to inhibit viral replication by targeting specific interactions between a virus and its host. BBR binds to DNA, inhibiting DNA synthesis and reverse transcriptase activity. It was shown to inhibit the replication of HSV [217], HCMV [218], HPV [219], DENV [220], HIV [221], HCV [222], and SARS-CoV-2 [223]. BBR exhibited anti-viral effects on IAV both in vitro and in vivo [224]. BBR possess the ability to control the MEK-ERK, AMPK/mTOR, and NF-κB signaling pathways, which are all necessary for viral replication. Furthermore, BBR has been reported to enhance the host immune response, leading to viral clearance.

Protein phosphorylation is crucial in the infection cycle of many viruses [39], affecting cellular protein’s stability, activity, interaction with other proteins, and infectivity. Viruses like EBV [42], HCV [42], SARS-CoV-2, DENV [43], and others [44,45,46], rely on MAPK p38 for replication, suggesting that MAPK p38 inhibitors may exhibit broad-spectrum anti-viral activity.

Varghese et al. discovered that BBR significantly reduces MAPK activity. The p38 mitogen-activated protein kinases (p38), extracellular signal-regulated kinases (ERK), and JNK signaling pathways are all significantly blocked by BBR, which specifically targets the ERK signaling pathway, resulting in a significant decrease in virion production. The reduction in viral protein expression following BBR treatment is most likely due to a decrease in virus-induced signaling. BBR treatment has no effect on virus entry or viral replicas’ enzymatic activity [225].

Additionally, it has been shown that BBR has the ability to suppress p38 MAPK activity in the context of HBV infection. The virion of HBV comprises a genome consisting of partially double-stranded relaxed circular DNA (rcDNA). Upon infecting a cell, this rcDNA is transformed into covalently closed circular DNA (cccDNA) in the nucleus. MAPK p38 activity plays a crucial role in the preservation of HBV covalently closed circular DNA (cccDNA) within infected cells [226]. The cccDNA functions as a molecular scaffold for the transcription of RNA molecules, such as mRNAs and pregenomic RNAs (pgRNAs). In the course of HBV’s life cycle, RT facilitates the conversion of the pregenomic RNA (pgRNA) into a partially double-stranded form of viral DNA known as relaxed circular DNA (rcDNA) within the viral capsid. The suppression of p38 MAPK activity has been associated with a decrease in the synthesis of HBV surface antigen (HBsAg), secretion of HBV e-antigen (HBeAg), and replication of HBV. The potential of BBR to effectively suppress the activity of MAPK presents a promising opportunity for the development of a novel antiviral drug targeting HBV infection.

### 2.5. Betulinic Acid

Betulinic acid (BA) is a naturally occurring pentacyclic triterpenoid found in the bark of various plant species, most notably the white birch (*Betula pubescens* Ehrh.) [227]. BA is capable of inducing apoptosis in tumor cells by directly activating the mitochondrial apoptosis pathway via a p53- and CD95-independent mechanism [228]. A computational approach demonstrated that BA has the capacity to alter HDAC6 and HDAC10 activities [229]. Furthermore, BA exhibited an anti-cancer activity that is mediated through cannabinoid receptors (CBs). BA functions as both a CB1 antagonist and a CB2 agonist [230].

BA was used for the treatment of various viral diseases [157]. BA has demonstrated activity in inhibiting DENV-2 NS5 polymerase [231]. Furthermore, BA exhibited an inhibitory effect on HBV replication [232]. Interestingly, the C-3 esterification of BA led to the discovery of Bevirimat, an HIV-1 maturation inhibitor patented by Sanofi-Aventis.

### 2.6. Butyric Acid

Butyric acid is a fatty acid derived from multiple vegetable sources that have anticancer activity through several pathways, including its influence on epigenetic machineries. Butyrate, alone or in combination with other drugs, including nicotinamide (NA), was shown to have anticancer activity in vivo [233]. Butyric acid exerts its anti-tumor effect by increasing HDAC expression and activity, which is accompanied by an upregulation of miR-203 promoter methylation [233].

Butyrate inhibited HBV replication and cell proliferation by inhibiting SIRT-1 expression in hepatoma cells. Specially, butyrate inhibited HBx protein expression, HBV-DNA, and hepatitis B surface antigen (HBsAg) [234].

### 2.7. Cardamonin

Cardamonin (CDN) is a natural chalcone isolated from the seeds of *Alpinia katsumadai Hayata* [235]. CDN has been shown to have a variety of pharmacological activities, including anticancer and anti-inflammatory properties [236]. It was recently revealed that CDN has anti-viral activity against the human coronavirus HCoV-OC43. CDN exhibits significant efficacy in reducing HCoV-OC43-induced cytopathic effects. CDN suppressed HCoV-OC43 infection by promoting the p38 MAPK signaling pathway and having therapeutic potential against other human coronaviruses [236].

### 2.8. Cordycepin

Cordycepin is a nucleotide analog derived from *Cordyceps mushrooms* [237]. In SNU719 cells, cordycepin treatment enhanced BAF chromatin remodeling complex subunit 7A (BCL7A) methylation while suppressing demethylation [238]. Cordycepin promoted methylation at EBV genomic sites near its Fp/Qp promoters. These findings indicate that cordycepin enhances DNMT3 activation, hence increasing the methylation of both genomic and EBV DNA loci in SNU719 cells [238], causing reduced EBV replication [239]. Cordycepin was also shown to have anti-SARS-CoV-2 replication activity [239].

Cordycepin shows anti-viral activities that are attributable to its ability to inhibit several protein kinases [237]. Cordycepin, an adenosine derivative, differs from adenosine in that its ribose lacks an oxygen atom in the 3′ position [240]. Several research groups have reported that cordycepin has anti-viral activity against several viruses including IAV, plant viruses, HIV, murine leukemia virus, EBV [241,242,243,244,245], and COVID-19 [246].

### 2.9. Corosolic Acid

Corosolic acid (CA) is a triterpene acid isolated from *Lagerstroemia speciose* L. [247]. This bioactive molecule is prevalent in foods such as guava, loquat, and olive, and has anti-inflammatory, anti-metabolic syndrome, and anti-neoplasic properties [248]. CA is implicated in the regulation of DNA methylation and histone H3 methylation. CA modulates CpG methylation sites, resulting in altered gene expression in treated cells [249]. Furthermore, CA inhibits the production and activity of epigenetic modulatory proteins, suggesting its capacity to prevent prostate carcinogenesis [250]. Furthermore, CA significantly increased the expression of acetylated histone H3 lysine 27 (H3K27ac) at the Nrf2 promoter, while decreasing histone H3 lysine 27 trimethylation (H3K27Me3) [251]. Moreover, its anti-viral activity against a number of viruses has been reported [248].

### 2.10. Curcumin

Curcumin, the major bioactive in turmeric, is a polyphenol with anti-inflammatory and anti-cancer activities [252]. Curcumin has been demonstrated to be a powerful epigenetic regulator with multiple effects on HDAC expression and activity. Curcumin decreased the expression of HDAC1, HDAC3, and HDAC8 proteins, as well as histone acetyltransferase p300, while enhancing the acetylation of Ac-histone H4 protein [253]. Curcumin was shown to reduce HAT activity and has been proposed as a potential DNMT and HDAC inhibitor [254].

Curcumin reduced the amount of HBsAg and the number of cccDNA copies, resulting in the inhibition of HBV replication, which was accompanied by a decrease in the acetylation level of cccDNA-bound histone H3 and H4 [255]. An MiRNA array revealed that miR-350, miR-17-2-3p, let 7e-3p, miR-1224, miR-466b-1-3p, miR-18a-5p, and miR-322-5p were downregulated by curcumin while miR-122-5p, miR-3473, miR-182, and miR-344a-3p were upregulated [256]. Overall, the curcumin-modified miRNAs had an impact on a number of signaling pathways, such as Wnt, NK-κB, MAPK, inflammatory response genes, and viral transmission [257].

**Figure 5:**
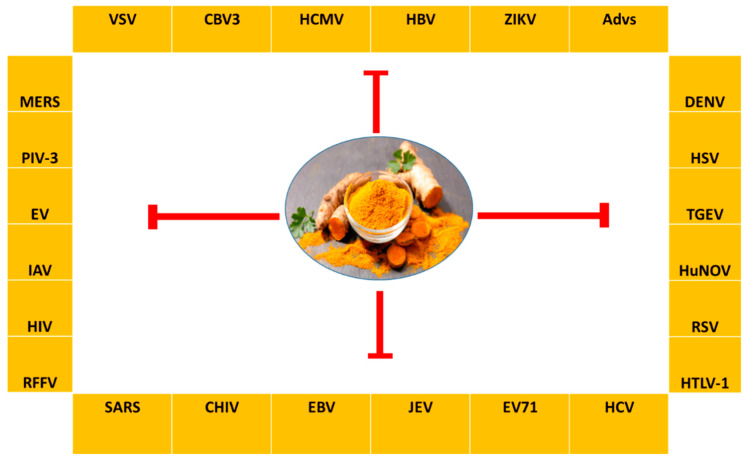
Anti-viral activity of curcumin. The figure shows the broad spectrum of antiviral activity of curcumin, which attacks most of the viruses studied.

Studies have shown that curcumin can inhibit the replication of various viruses, including HBV [255], HIV [258], and IAV [259]. It exerts its anti-viral effects by interfering with viral replication processes and by modulating the epigenetic regulation of genes involved in viral infection [255]. *Curcuma longa* L. (CLL) extract inhibits the transcription of the HBV X (HBx) gene through a p53-mediated pathway, with no cytotoxicity to liver cells. These results highlight CLL extract as an efficient natural product with anti-HBV activity [260]. Curcumin interfered with the binding activity of activator protein-1 (AP-1) in HeLa cells, resulting in the decreased transcription of HPV-18 genes [261]. The transcription factor AP-1 regulates epithelial tissue-specific gene expression in almost all HPV types. Because it changes apoptosis and lowers viral genes, these results suggest that curcumin may be a good choice for treating highly oncogenic HPV infections. Curcumin may also work against viruses by changing how viruses enter cells, how viral protease enzymes work, and how host cells communicate with each other [262]. Curcumin and its analogs could also stop the growth of ZIKV, CHIKV, VSV, CVB3, EV71, RSV, HSV-2, KSHV, and HAdV [263]. Curcumin has been shown to either directly or indirectly stop the replication of viruses by changing the immune response of the host, which leads to the removal of the viruses [264] (Figure 5).

Curcumin is known to strongly stop the production of several proinflammatory cytokines that cause the “cytokine storm” that occurs in some viral infections. New in silico docking studies have confirmed that curcumin may be a good candidate for helping with the acute symptoms of SARS-CoV-2 infection [265]. Moreover, a human study in Pakistan evaluated the efficacy and safety of an oral curcumin-quercetin supplement plus standard care vs. standard care alone in outpatients with early-stage COVID-19. The study found that the curcumin-quercetin group had a significantly higher viral clearance (18/25 (72.%)) compared to that of the control group [266]. In addition, curcumin-quercetin reduced acute COVID-19 symptoms in a group compared to a control group [266].

### 2.11. Ellagic Acid

Ellagic acid (EA) is a ubiquitous phenolic molecule isolated from a variety of fruits and vegetables and is well known for its anti-cancer effect [267]. This bioactive substance has been demonstrated to effectively induce HDAC activity. Human adipogenic stem cells treated with EA showed a substantial increase in HDACs’ gene expression. EA also suppresses adipocyte differentiation through coactivator-associated arginine methyltransferase 1 (CARM1)-mediated chromatin modification. This compound also inhibited adipocyte growth and differentiation by increasing histone arginine methylation [268], resulting in an increase in acetylated histone through epigenetic alterations mediated by coactivator-associated CARM1 inhibition. CARM1 inhibition was shown to limit H3R17 methylation, resulting in decreased H3K9 acetylation and HDAC9 dissociation [268]. Ellagic acid and other plant-derived substances strongly bound with the multiple targets of the SARS-CoV-2 receptors, inhibiting viral entry, attachment, binding, replication, transcription, maturation, packaging, and spread [269].

### 2.12. Epigallocatechin Gallate

Epigallocatechin gallate (EGCG) is the most abundant catechin in tea leaves, comprising 50–80% of the total catechins [270]. EGCG was recognized as the primary contributor to the numerous health benefits associated with green tea [270], including a reduction in the symptoms of infectious diseases [271].

EGCG binds to various targets and exerts its influence on the activity of diverse enzymes and signal transduction pathways [272]. Studies with animal models and various cancer cell lines have shown that EGCG and other catechins modulate the activity of DNMTs [273]. Fang et al. suggested that EGCG inhibited DNMT activity, resulting in the reactivation of methylation-silenced genes [274]. In fact, EGCG can reduce DNA methylation through the direct inhibition of the activity of DNMT 1, DNMT 3a, and DNMT 3b, by directly binding to the active site within the enzyme [273]. In contrast, Lee et al. provided an alternative mechanism to explain the DNMT inhibition induced by EGCG. Their studies revealed that DNA methylation was primarily inhibited in vitro through competitive inhibition by promoting the formation of SAH (S-adenosylhomocysteine). Based on this mechanism, EGCG causes a reduction in the intracellular concentration of SAM (S-adenosylmethionine), which is the universal methyl donor, while simultaneously increasing the concentration of SAH. Importantly, SAH acts as a feedback inhibitor for various methylation reactions that depend on SAM. Thus, the modulation of SAM and SAH levels by EGCG contributes to the inhibition of DNMT activity and other SAM-dependent enzymes such as methyltransferases (MTases) [273,275,276].

EGCG also regulates histone modifications by inhibiting the activity of HDACs [275] and consequently inducing changes in gene expression patterns. The inhibition of HDAC activity by EGCG results in a decrease in HDAC enzyme activity and consequently leads to increased levels of acetylation on histone proteins both globally and at specific regions. In human colon cancer cell lines, EGCG inhibited HDAC1, HDAC2, and HDAC3 expression [277]. In addition, EGCG inhibited HAT activity [278]. EGCG has been demonstrated to prompt the increased acetylation of lysine 14 and 9 (on histone H3) and lysine 12, 5, and 16 (H3-Lys and H4-Lys) levels [279]. In addition, EGCG can increase the acetylation of histones H3K9/14ac and H3ac, as well as the concomitant hypermethylation of active H3K4me3 and restrictive H3K9me3 chromatin proteins [280]. Furthermore, in cancer cell lines, EGCG inhibits DNMT’s activity and reactivates methylation-silenced genes, such as p16INK4a and retinoic acid receptor beta (RARβ) [281]. Moreover, a recent report showed a potential effect of EGCG in modulating NAD+ levels and thereby the activity of SIRT proteins [282].

EGCG has also been implicated as a potential modulator of miRNAs by regulating the expression levels of epigenetic modifiers or viral proteins. EGCG has been reported to decrease the levels of let-7e-5p, miR-103a-3p, miR-151a-5p, miR-195-5p, miR-222-3p, miR-23a-3p, miR-23b-3p, miR-26a-5p, miR-27a-3p, miR-29b-3p, miR- 3195, miR-3651, miR-4281, miR-4459, miR-4516, miR-762, and miR-125b-5p [283]. Another study showed that EGCG enhances the expression of miR-3663-3p, miR-1181, miR-3613-3p, miR1281, and miR-1539, while decreasing miR-221-5p, miR-374b, miR-4306, miR-500a-5p, and miR590-5p in human dermal papilla cells [284] and miR-140-3p and miR-221 in melanoma and hepatoma cell lines, respectively [285,286]. The anti-viral properties of EGCG have been demonstrated for a wide range of virus families, including Retroviridae, Orthomyxoviridae, and Flaviviridae. Furthermore, the molecule affects the replication cycle of DNA viruses such as HBV, HSV, and AdV [287]. Particularly, EGCG has been shown to inhibit the replication of several viruses, including IAV, HBV, HCV, HSV-1 and HSV-2, HPV, ZIKV, and SARS-CoV-2 [288,289,290,291,292,293,294]. EGCG showed an anti-proliferative effect in the HPV-16-associated cervical cancer cell line CaSki via the arrest of the cell cycle in the G1 phase, leading to programmed cell death [295]. Interestingly, EGCG and other catechins from green tea are effective against HPV-mediated lesions of the cervix. The US Food and Drug Administration (FDA) has approved Veregen^®^, a topical ointment with 15% green tea extract or sinecatechins, to treat genital warts that are caused by HPV infection [292,296].

EGCG also exerts anti-viral activity by modulating miRNA expression, such as upregulating miR-548m expression. Reports found miR-548m binding sites in the 3′UTR of CD81 mRNA′. This suggests that miR-548m may lower the expression of CD81, which would make HCV less likely to infect cells. These results suggest that EGCG may act as an anti-HCV drug by increasing the expression of miR-548m while decreasing the expression of the CD81 receptor required for HCV infection [291]. In addition, miR-194 has been reported to prevent HCV entry by targeting CD81 receptors [297].

**Figure 6:**
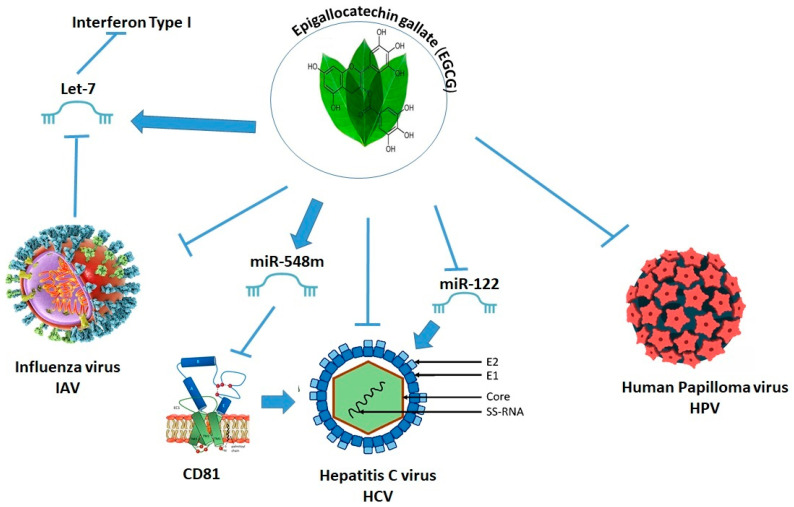
Anti-viral activity of EGCG against IAV, HPV, and HCV viruses. HCV replication dependent on host CD81 and host miR-122. EGCG upregulated miR-548m expression, which in turn regulates CD81 expression and downregulates miR-122 (also mediated by other bioactive substances such as resveratrol) to exert anti-HCV activity. IAV infection caused a significant decrease in microRNA let-7 expression which is required for regulating expression of type I interferon. EGCG and other bioactive substances, such as quercetin, upregulate let-7 to increase interferon expression and effectively inhibit IAV infection.

The liver-specific miR-122 [112] is the most abundant miRNA in the liver, accounting for 60–70% of the total miRNA in hepatocytes. Many investigations have found that miR-122 is required for HCV replication in infected cells [113,114,115]. EGCG (and also resveratrol) modulates the expression levels of miR-122 and thus might exert an anti-HCV effect via this mechanism. IAV infection caused a significant decrease in micro-RNA let-7 expression in host cells that normally regulate the expression of type I interferon required for the host cells’ anti-viral activity. The overexpression of let-7 increased the expression of the interferon and effectively inhibited the IAV infection. EGCG upregulates the expression of let-7 and thereby has the potential to exhibit anti-influenza activity [202] (Figure 6).

### 2.13. Galangin

Galangin is a naturally occurring flavonoid found in honey that is also an active ingredient in galangal, a spice used in traditional Chinese medicine [298]. This natural compound appears to effectively inhibit HDAC activity. In SH-SY5Y human neuroblastoma cells, treatment with galangin increased endogenous HDAC1-mediated deacetylation independently of DNA methylation status and subsequently decreased histone H3 acetylation in BACE1 promoter regions [299].

Galangin upregulates miR-455-5p to modulate the regulatory subunit 12A of protein phosphatase 1 (PPP1R12A). This effect suppresses the activation of the MAPK and the phosphoinositide 3-kinases/protein kinase B (PI3K/AKT) pathways, controlling cancer cell survival and metastasis [300]. Galangin showed significant antiviral activity against HSV-1 and CoxB1 [301].

### 2.14. Garcinol

Garcinol is a polyisoprenylated benzophenone isolated from the peel of the *Garcinia indica* Choisy fruit [302]. Garcinol has anti-cancer, anti-inflammatory, and antioxidant properties [101]. In tumor cells, it primarily inhibits the NF-κB and Janus kinase (JAK)/STAT3 transcription factors [302]. Garcinol has been shown to decrease the HAT activity of p300 and pCAF in vitro and in vivo [303]. As a result, garcinol was discovered to be a potent inducer of apoptosis and to affect global gene expression in HeLa cells [303]. The chemical structure of garcinol shows some similarities with curcumin (β-diketone, phenol). Garcinol has shown significant anticancer activity by targeting NF-κB, 5-lipoxygenase (5- LOX), and STAT proteins [304,305]. In addition, garcinol is a well-documented HAT inhibitor and thus plays an important role in the epigenetic regulation of gene expression [306]. Garcinol is utilized in Ayurvedic medicine for the treatment of infections and edema [307,308]. Garcinol downregulated miR-21, miR-494, miR-495, and miR-1977 in pancreas cancer cells [309] and upregulated the expression of miR-453, miR-128, miR-1280, and miR-720 [310]. In breast cancer cells, garcinol can induce the expression of the tumor suppressor miRNAs let-7a, let-7e, let-7f, miR-200b, and miR-200c both in vitro and in vivo [311]. A recent report provided evidence for the ability of garcinol to inhibit HIV-1 reverse-transcriptase-associated ribonuclease H [312].

### 2.15. Genistein

Genistein is a naturally occurring isoflavone isolated from the plant *Genista tinctoria* [313] and is well known for its potential chemotherapeutic action against a variety of cancer cells. Studies on HAT and HDAC activity revealed that genistein reduces HDAC while increasing HAT activity [313]. In prostate cancer cell lines, a chromatin immunoprecipitation analysis with multiple antibodies revealed the enrichment of acetylated histones H3, H4, and H3 di- and tri-methylated lysine 4 after incubation with genistein [314]. Furthermore, genistein inhibited the phosphorylation of serine 10 and the methylation of lysine 9 in the promoter regions of several genes, including wingless-related integration site (Wnt5a), as well as induced the secretion of frizzled-related protein 5 (Sfrp5), and frizzled-related protein 2 (Sfrp2) [315]. Moreover, the genistein treatment significantly inhibited miR-223 expression and upregulated the F-box and WD repeat domain-containing 7 (Fbw7) proteins which act as a tumor suppressor gene. Moreover, the downregulation of miR-223 inhibited cell growth and induced apoptosis in PC cells [316]. In addition, other miR-223 targets, such as granzyme B, IκB kinases (IKKs), and signal transducer and activator of transcription 3 (STAT3), are expected to be affected by genistein and thereby modulate immune response [116]. Interestingly, miR-223 expression is downregulated during IAV, HBV [116], HCV, HIV [115], and SARS-CoV infections [117]. Thus, it is reasonable to speculate that the antiviral activity of genistein might be mediated by the regulation of miR-223.

### 2.16. Ginkgolic Acid

Ginkgolic acid (GIA) is a phenolic acid found in *Ginkgo biloba* L. with neuroprotective, antimicrobial, and antitumor properties [317]. Ginkgo biloba has been used in traditional Chinese medicine since at least the 11th century BC to treat various ailments, such as dementia, asthma, bronchitis, and kidney and bladder diseases. Ginkgolic acid is a potent multitarget inhibitor of key enzymes in the biosynthesis of proinflammatory substances [317].

Ginkgolic acid impairs SUMOylation by blocking the formation of an E1-SUMO thioester complex by binding directly to E1 [318]. SUMOylation is a process by which small ubiquitin-related modifier proteins (SUMO) covalently bind to specific lysine residues in target proteins, thereby regulating various aspects of protein function, including transcription, subcellular localization, DNA repair, and the cell cycle [319].

JMJD2A, a member of the Jumonji domain 2 (JMJD2) family, is the histone demethylase responsible for the accumulation of SUMO-2/3. JMJD2A is SUMOylated at lysine 471 by Kaposi’s sarcoma-associated herpesvirus (KSHV) K-bZIP, a viral SUMO-2/3-specific E3 ligase, in a SUMO-interacting motif (SIM)-dependent manner. SUMOylation is required for the stabilization of chromatin association and gene transactivation by JMJD2A [320]. Recently, ginkgolic acid was reported to inhibit HSV-1 by disrupting the virus’ structure, blocking fusion, and inhibiting viral protein synthesis [321]. Moreover, ginkgolic acid is a powerful antiviral that can inhibit three types of fusion proteins, such as those from HIV, EBOV, IVA, and EBV. Moreover, ginkgolic acid inhibited HIV protease activity in a concentration-dependent manner. In addition, treatment with ginkgolic acid inhibited HIV infection in PBMCs in a concentration-dependent manner [322].

### 2.17. Glycyrrhizic Acid

Glycyrrhizic acid (GA) is a triterpene isolated from the roots and rhizomes of licorice (*Glycyrrhiza glabra* L.)) [323]. GA is the principal bioactive ingredient of licorice with anti-viral [324], anti-inflammatory, and hepatoprotective effects [325]. The licorice plant is native to Europe and Asia and has been used for centuries in traditional medicine. Ancient documentations from China, India, and Greece stated that it was traditionally used to alleviate the symptoms of viral respiratory tract infections and hepatitis [323]. Licorice is known for its ability to inhibit the viral replication of various viruses including HBV, HCV, IAV H1N1, and HIV, as reviewed by Zhong et al. [326]. Licorice extract containing glycyrrhiza inhibited the replication of Newcastle disease virus (NDV) and was non-toxic in an embryonic egg assay [327]. Glycyrrhizin exhibited antiviral activity by affecting cellular signaling pathways and increasing the expression of nitrous oxide synthase (NOS) [328]. In vitro studies revealed it also has anti-viral activity against SARS-related coronavirus, respiratory syncytial virus (RSV), arboviruses, vaccinia virus (VACV), and vesicular stomatitis virus (VSV) [329,330]. In animal studies, treatment with glycyrrhizin reduced mortality and the viral activity of HSV, encephalitis, and IAV pneumonia [329]. In addition to its antiviral effect, glycyrrhizin also showed anti-inflammatory effects caused by the decreased release of IL -6 from macrophages, resulting in a reduced induction of cytokine storms [331]. In addition, high concentrations of licorice in RAW264.7 mouse macrophage cells inhibited LPS-induced nitric oxide production in a concentration-dependent manner [332].

Presumably by controlling the expression of the NF-κB and PI3K signaling pathways, glycyrrhizin’s anti-inflammatory impact may be obtained [333]. Glabridin licorice (*Glycyrrhiza glabra*) contains significant amounts of the isoflavan glabridin, which demonstrated anti-inflammatory and neuro- and cardioprotective activities in addition to distinct anti-cancer properties (growth inhibition as well as anti-angiogenic and anti-metastatic effects [334,335,336]. Glabridin suppressed cancer stem-cell-like features in hepatocellular carcinoma cells by the upregulation of miR-148a that targets SMAD2 (mothers against decapentaplegic homolog 2) associated with the inhibition of TGF (transforming growth factor)-β/SMAD2 signaling [337,338]. Interestingly, miR-148 also targets DNMT3b [84]. In addition, glabridin inhibited the angiogenesis of breast tumors by the upregulation of miR-520a [339]. The expression of NF-κB was blocked by the upregulation of miR-520a in glabridin-treated breast cancer cells, which was accompanied by the inhibition of NF-κB/IL-6/STAT-3 signaling. Host signaling pathways regulated by glabridin are essential for viral infections and viral diseases. Glycyrrhizic acid exhibited anti-viral activity [340,341]. It inhibits HSV-1, HSV-2, VZV, HCMV, ZIKV, IAV, EBV, HIV, EBOV, and SARS-CoV-2 by varying mechanism of actions [342].

### 2.18. Grifolin

Grifolin is an adenosine derivative isolated from the fresh fruiting bodies of the fungus *Albatrellus confluens* (Alb. & Schwein.) Kotl. & Pouz. [343]. Grifolin was shown to suppress tumor cell lines’ proliferation. Grifolin inhibited Bcl-2 expression while increasing Bax expression [343]. Grifolin reduced Elk1 transcription as well as its binding to the DNMT1 promoter region. The mRNA levels of pTEN and Timp2 are likewise increased by griforolin. Grifolin’s anti-tumor effects may be exerted by ERK1/2-Elk1-DNMT1 signaling’s epigenetic activation of metastasis-inhibitory genes [344].

### 2.19. Oleacein

Oleacein, a secoiridoid [345], is the most prominent phenolic compound in Olea europaea L. (olive). This substance exhibited anti-cancer activity against multiple myeloma cell lines (NCI-H929, RPMI-8226, U266, MM1s, and JJN3) and was found to be an effective epigenetic modulator. Oleacein was reported to downregulate several class I/II HDACs both at the mRNA and protein level; conversely, no effect on global DNA methylation was associated with this compound [346,347]. Oleacein inhibited the proliferation of numerous melanoma cell lines [348]. It has been shown that oleacein can stop HIV-1 infection, replication, and the production of the viral core antigen p24 [349].

### 2.20. Organosulfur Chemicals

Organosulfur chemicals (OSC) are a group of compounds found in garlic (*Allium sativum* L.). More than thirty sulfur-containing compounds have been identified so far [350]. Garlic extracts were found to have broad-spectrum anti-viral activity [351]. Conversely, the mechanism by which these extracts or their purified constituents exert anti-viral activity may differ depending on the virus strains and viral lifecycle, which includes viral entry, fusion, replication, assembly, and virus–host-specific interactions [352]. Furthermore, one of the possible activities of garlic extracts and bioactive moieties that may combat viral infections resides in its immunomodulatory properties.

Garlic has been used as an ethnomedicinal herb to cure infectious diseases for ages [353]. It has been utilized to treat a variety of illnesses in African traditional medicine, including sexually transmitted diseases, Mycobacterium tuberculosis (TB), respiratory tract infections, and wounds [354,355]. Garlic was shown to have effects against viral infections in humans, animals, and plants. In addition to garlic extracts or powders, purified bioactive components from garlic also exhibited anti-viral activity. As an example, alliin (S-allyl-L-cysteine sulfoxide), which is the most abundant sulfur compound found in fresh and dry garlic [356], is rapidly converted into allicin (diallyl thiosulfinate) by alliinase enzymes when fresh garlic is chopped, minced, crushed, or chewed [356,357]. Allicin is the primary component responsible for its anti-viral activity [358,359], immunomodulatory characteristics [360], anti-inflammatory [361] and antioxidant [362] activities, and other pharmacological properties. Allicin is very unstable and breaks down into other OSCs, such as andajoen, vinyl dithiins, diallyl disulfide (also known as garlicin or DADS), diallyl trisulfide (also known as allitridin or DATS), and diallyl disulfide (also known as DAS). In vivo, allicin can interact with cellular thiols such as glutathione and L-cysteine to form S-allyl mercapto glutathione (SAMG) and S-allyl mercaptocysteine (SAMC) [363,364]. These compounds may be responsible for structural changes in pathogen proteins [357]. Preclinical in vitro and in vivo studies have shown that allicin-derived OSCs such as ajoene, allitridine, garlicin, and DAS have antiviral [365,366,367,368,369], immunostrengthening [370,371,372], and other therapeutic activities [363,373,374].

### 2.21. Orobol 7-O-d-Glucoside

Orobol 7-O-d-glucoside (O7G) isolated from banaba *Lagerstroemia speciosa* L. (Lythraceae) [375] was tested for its anti-viral efficacy against eight different strains of HRV, a cause of common viral respiratory tract disease [375]. O7G displayed anti-viral activity against HRV A and B, as well as species resistance to pleconaril, a potent capsid inhibitor of HRVs [376].

### 2.22. Orsaponin

Orsaponin (OSW-1) is a natural substance derived from the bulbs of the plant *Ornithogalum saundersiae* which has anti-proliferative and anti-cancer properties [377]. Enteroviruses (EV) use oxysterol-binding protein (OSBP) as a host lipid transport protein [378]. Several studies have shown that OSW-1 binds to one of the two identified OSBP ligand-binding sites and exerts prophylactic antiviral activity against all enteroviruses tested, including EV71, coxsackievirus A21 (CVA21), and HRV-B [379,380].

### 2.23. Plitidepsin

Plitidepsin is a cyclic depsipeptide isolated from the Mediterranean marine tunicate *Aplidium albicans* [381]. Plitidepsin is made and sold as alpidine, a drug that has been approved for a limited number of uses to treat multiple myeloma. Its target is eukaryotic translation elongation factor 1A (eEF1A) [382]. This cellular component is necessary for enzymes to move aminoacyl-tRNAs to the ribosome. It has also been found to be an important host factor in the replication of many viruses, such as RSV and gastroenteritis coronavirus [383]. In an in vitro and in vivo investigation, White and colleagues discovered that plitidepsin had anti-viral efficacy against SARS-CoV-2 via inhibiting eEF1A [384]. Plitidepsin was shown in vitro to be 27.5 times more potent than remdesivir in Vero E6 cells. In two animal models, plitidepsin treatment lowered SARS-CoV-2 replication and protected against the SARS-CoV-2 B.1.1.7 variant as well as lung inflammation [384,385]. A phase I/II clinical trial for the use of plitidepsin in the treatment of COVID-19 (NCT04382066) was conducted.

### 2.24. Pterostilbene

Pterostilbene (3,5-dimethoxy-4-hydroxystilbene) is a bioactive chemical found in grapes and several berries, mainly blueberries [386]. Pterostilbene altered gene expression in breast cancer cells, which are mediated by epigenetic mechanisms such as HDAC modifications [387]. It inhibits SIRT1 and regulates cell proliferation, apoptosis, stress response, metabolism, cellular senescence, and cancer [387,388].

Interestingly, a recent report demonstrated that resveratrol and pterostilbene inhibit SARS-CoV-2 replication in human primary bronchial epithelial cells [389].

### 2.25. Quercetin 

Quercetin is a flavonoid found in many medicinal plants and food products [390]. This compound has a variety of biological properties, including anticancer activity, through several modes of action. Quercetin, alone or in combination with other drugs, promotes epigenetic modifications. It enhances histone H3 acetylation via FasL overexpression, the activation of HAT, and the inhibition of HDAC activities [391]. Furthermore, quercetin reduced HMT activities, particularly HMT-H3K9 activity [213]. In addition, quercetin was reported to reduce the expression of miRNA, such as that of miR-146a [392], a regulator of HIV replication [393], and NF-κB signaling which is associated with anti-inflammation activity [394]. MiR-16, miR-217, and miR-145 were also modulated by quercetin [395,396,397].

Quercetin was shown to inhibit the replication of several viruses, including IAV H1N1, IVA H3N2, HBV, HCV, DENV, poliovirus, rhinovirus, CHIKV, MERS-CoV, HSV 1/2, EBV, RSV, Arbovirus, EBOV, HIV, Japanese encephalitis virus (JEV), hAdV, enterovirus (EV), ZIKV, NDV, Mayaro virus (MAYV), and SARS-CoV-2 [197,293,398,399,400]. It can modulate DNA methylation and histone acetylation [401]. Moreover, it has been reported to activate SIRT1 and exhibit anti-viral effects against several viruses [402]. Quercetin attenuates HCV production [403] and inhibits the propagation of HSV-1 [398]. IAV infection causes a significant decrease in microRNA let-7 expression in host cells that normally regulate the expression of type I interferon required for host cells’ anti-viral activity. Quercetin upregulates the expression of let-7 and thereby has the potential to exhibit anti-IVA activity [202].

### 2.26. Raoulic Acid

Raoulic acid isolated from *Raoulia australis* [133] has shown possible anti-viral activity against coxsackievirus B3 (CVB3) and coxsackievirus B4 (CVB4), as well as HRV types A and B [133].

### 2.27. Resveratrol

Resveratrol (3,5,4′-trihydroxystilbene) is a bioactive molecule isolated by Saiko et al. [404] from the roots of white hellebore (*Veratrum grandiflorum* Loes.). More than 50 plant species contain this bioactive substance, including grapes, apples, blueberries, plums, and peanuts. It has been intensively researched for its health benefits against a variety of diseases, including cancer [405]. Resveratrol treatment increased p21 expression in Caski cells via the inhibition of HDAC [406]. HDAC activity is decreased by resveratrol in a dose-dependent manner [407]. Pterostilbene is a phytoalexin dimethyl ether molecule that is a dimethoxylated derivative of resveratrol [408]. Interestingly, resveratrol is known to activate SIRT1 [409]. Despite the fact that research into the potential of SIRT1 activators’ anti-viral activities is continuously being conducted, it is important to keep in mind that this field is still in its infancy and that specific natural compounds that directly activate SIRT1 and have anti-viral effects are not yet well established.

Over 100 scientific documents have implicated miRNAs in resveratrol’s health-promoting activity. For example, in human colon cancer cells, resveratrol significantly decreased the levels of miR-17, miR-21, miR-25, miR-92a-2, miR-103-1, and miR-103-2 [410]. Meanwhile, in lung tumors, resveratrol led to an upregulation of miR-200c [411].

Resveratrol intake in humans for six months increased miR-21, miR-181b, miR-663, and miR-30c, while reducing inflammatory cytokines like IL-6, CCL3, IL-1β, and TNF-α. This reduction was mediated by the TLR and NF-κB signaling pathways [412].

Resveratrol showed anti-viral properties [413]. It inhibit HSV infection in vitro and in vivo [414], but also inhibit beta-corona viruses such as MERS-COV and SARS-CoV-2 [415]. Resveratrol inhibited SARS-CoV-2 replication in Vero cells and Vero E6 cells, with IC_50_ values of 4.48 μM and 11.42, respectively [416]. It also inhibited SARS-CoV-2 Mpro activity, suggesting resveratrol as a potential therapeutic target [417].

An HSV-2 infection was regulated by resveratrol-induced increased histone acetylation [418]. Varicella-zoster virus (VZV) replication in vitro was reduced by resveratrol in a dose- and time-dependent manner. The inhibited activation of the IE62 gene by resveratrol was accompanied by a reduction in infections of both wild-type and DNA polymerase mutants with acyclovir-resistant VZV [419,420]. Furthermore, resveratrol exhibits activity against VEEV [421], EBV [422], CBV [423], and RSV. Resveratrol was shown to regulate TLR3 expression, inhibit the TIR domain containing adaptor molecule (TRIF) signaling pathway, and induce M2 receptor expression following RSV infection [424]. There is growing evidence showing that HBV can alter the expression levels of all SIRT proteins, an NAD+-dependent deacetylate. All SIRTs, in turn, regulate HBV replication through a cascade of molecular mechanisms. In addition, several studies suggest that targeting SIRTs with appropriate drugs is a potential treatment strategy for HBV infection [425]. Resveratrol also stops the replication of RSV in human bronchial epithelial cells by activating SIRT1 and increasing the release of TNF-α, which promotes cell death [402].

### 2.28. Silibinin

Silibinin is a flavonolignan derived from milk thistle [426] and has powerful anticancer effects, targeting multiple checkpoints, including epigenetic processes such as HDAC activity. Silibinin was shown to inhibit the expression of HDAC2 and HDAC3 proteins, as well as HDAC1, HDAC6, SET domain proteins (SETD1A, D4, D6), and lysine-specific demethylases (KDM 5B, 5C, and 4A) are some of these [315]. Silibinin also inhibited the expression of HDAC1-2 in DU145 and PC3 human prostate cancer cell lines [56].

Enhanced Lys27 trimethylation on H3 (H3K27me3) [427], the inhibited activity of DNMTs, and increased global DNA hypomethylation [428,429,430] were also caused by silibinin in different test systems. Moreover, silibinin decreased the expression of miR-21 and miR-155 [431]. Recent studies have documented the antiviral activities of silibinin against several viruses, including the flaviviruses (HBV and DENV), togaviruses (CHIKV and MAYV), IVA, HIV, and HBV [432].

### 2.29. Silvestrol

Silvestrol, isolated from *Aglaia* plants [433], has been shown to target eukaryotic initiation factor-4A (eIF4A), an RNA helicase whose activity is required to unravel RNA secondary structures in the 5’-untranslated region (5′-UTRs) and facilitate translation initiation [434]. Silvestrol showed activity against EBOV, ZIKV, CHIKV, and coronaviruses [435,436,437]. A study by Mueller and others [438] found that silvestrol stopped the translation of MERS-CoV and HCoV-229E viral mRNA in MRC-5 human embryonic lung fibroblasts. Furthermore, silvestrol stops the production of MERS-CoV structural and nonstructural proteins (N, nsp8) and the creation of viral replication and transcription complexes in PBMCs [438]. More research has shown that the synthetic rocaglate CR-31-B (-) can stop the replication of HCoV-229E and SARS-CoV-2 in both in vitro and ex vivo settings [439,440].

### 2.30. Sulforaphane

Sulforaphane (1-isothiocyanato-4(methylsulfinyl)butane) (SFN) is an isothiocyanate present mostly in cruciferous vegetables including broccoli, cabbage, brussel sprouts, and radishes [441]. In breast cancer cells, SFN significantly reduced HDAC activity [442] and increased the expression of acetylated histones H3 and H4 [442]. Moreover, SFN enhanced the expression of the anti-oncogene proteins dual-specificity phosphatase 4 (DUSP4) and cyclin-dependent kinases (CDKs), which are associated with the downregulation of the HDAC5 and HDAC11 genes in the hepatocarcinoma HepG2 cell line [443]. A further benefit of SFN is that it increases let-7 expression, which may have anti-IAV effects [202]. Moreover, SFN inhibits HCV [444] and DENV replication by the enhancement of anti-viral interferon response through Nrf2-mediated HO-1 induction and the inhibition of DENV protease [445]. In addition, SFN inhibited the in vitro replication of six strains of SARS-CoV-2, including Delta and Omicron, and the seasonal coronavirus HCoV-OC43 [446]. SFN inhibited SARS-CoV-2 replication in vitro and in vivo, and when administered to K18-hACE2 mice, it significantly decreased the viral load, reduced lung injury, and reduced immune cell activation, suggesting its potential use as a prevention or treatment agent for coronavirus infections [446].

In addition, SFN and remdesivir interacted synergistically to inhibit coronavirus infection in vitro [447]. SFN treatment diminished immune cell activation in the lungs [446] possibly mediated by the overexpression of let-7 which regulates immune response in infected cells [202]. SFN anti-viral activity was exhibited both in vitro and in vivo by interfering with viral replications as well as modulating the inflammatory immune response and leading to a decreased viral load.

### 2.31. Tanshinone IIA

Tanshinone IIA is a natural bioactive compound found in *Salvia miltiorrhiza* Bunge’s rhizome [448]. Wang et al. investigated tanshinone IIA’s role in epigenetic modifications, demonstrating its effect on HDAC modification [449]. This bioactive molecule decreased the enzymatic activity of HDACs. Tanshinone IIA significantly reduced the protein levels of HDAC1, HDAC3, and HDAC8 by lowering mRNA expression [449].

Tanshinone IIA was reported to be an inhibitor of MAPK p38 [450]. MAPK p38 is explored by many viruses for their efficient replications [47]. Natural products that inhibit MAPK p38 activity might be a good candidate to exhibit broad-spectrum anti-viral activity [450], including DENV [43], coronavirus [44], VEEV [45], EV71 [46], SFTSV, HSV-1, and SARS-CoV-2 [47,450].

### 2.32. Ursolic Acid

Ursolic acid (3-beta-3-hydroxy-urs-12-ene-28-oic-acid) is a triterpenic acid found in ginseng (*Panax Ginseng* C. A. Meyer), rosemary (*Rosmarinus officinalis* L.), apple peel, pear, cranberry, and plum (*Prunus domestica* L.) [451]. It has been extensively studied for its chemopreventive and chemotherapeutic effects on a variety of malignancies. Ursolic acid significantly reduces the expression of various epigenetic regulatory factors, including HDAC1, HDAC2, HDAC3, and HDAC8 (Class I), as well as HDAC6 and HDAC7 (Class II) [452]. Zhao et al. (2012) reported an anti-CMV effect of ursolic acid which was significantly stronger than that of ganciclovir [453].

### 2.33. Withaferin A

Withaferin A (WFA) is a steroidal lactone derived from the plant *Withania somnifera* (L.) Dunal [454], known for its anticancer properties and ability to target several cancer hallmarks such as cell proliferation, migration, invasion, and angiogenesis, as well as the epigenetic process [454]. WFA displayed chemopreventive benefits by reversing epigenetic alterations via the downregulation of HDAC1 protein levels [454]. Furthermore, WFA, alone or in combination with SFN, significantly reduced HDAC1 expression at both the mRNA and protein levels [455]. WFA with SFN decreased the HMT activity, but enhanced the HAT activity [455].

WFA as well as other plant-derived substances exhibited a high potential in modulating the main protease (Mpro) activity and cytokine storm in COVID-19 infection [456]. Moreover, WFA has the potential to attenuate the neuraminidase (NA) of H1N1 IVA. The docking results of [456] predicted the high binding affinity of WFA toward NA [457].

## 3. Conclusions and Future Directions

**Figure 7:**
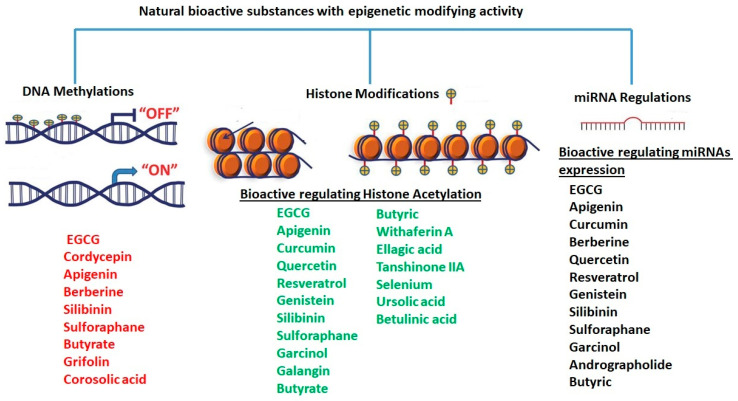
Natural bioactive substances with epigenetic modifying activity. Shows plant-derived bioactive compounds that modulate DNA methylation (**left**), histone medication (**middle**), and miRNA expression (**right**).

In this review, we narrowed our study to activity against 20 main viruses, and we reviewed 33 significant bioactive compounds (Table 3 and Figure 7) that had anti-viral and epigenetic-altering properties.

Not all bioactive substances that alter epigenetic modifications were reported to have anti-viral activity. On the other hand, other bioactive substances exhibited anti-viral activity without any evidence of epigenetic effects. This suggests that epigenetic pathways might contribute to the anti-viral effect of these bioactive compounds, but are not the exclusive mechanisms explaining their action. This missing information will need to be evaluated experimentally.

We have shown that a variety of bioactive compounds can modulate epigenetic modifications such as DNA methylation, histone modifications, and miRNA expression. Bioactive substances such as EGCG, apigenin, curcumin, quercetin, berberine, resveratrol, genistein, silibinin, and sulforaphane were particularly interesting for their complex effect on different epigenetic pathways. Some bioactive substances, such as ellagic acid, tanshinone IIA, selenium, cordyceptin, grifolin, andrographolide, ursolic acid, corosolic acid, and betulinic acid, can affect just one epigenetic mark, while others, such as EGCG, showed activities on all epigenetic features.

It should be noted that not all bioactive chemicals that influence the activity of epigenetic mediators have been evaluated for anti-viral activity. Others showed anti-viral efficacy but without a definite mechanism of action. Some bioactive substances showed anti-viral activity against a limited number of viruses, whilst others were efficient in inhibiting a wide range of viruses. Some bioactive substances have a broad-spectrum anti-viral activity, inhibiting both RNA and DNA viruses. Among all the compounds, curcumin and glycyrrhizic acid are especially notable. Glycyrrhizic acid inhibited 80% and 65% of the DNA and RNA viruses (respectively) reviewed. Curcumin also inhibited 70% and 55% of the DNA and RNA viruses (respectively) examined. Others have been reported to inhibit just a few viruses, which may be connected to the mechanism of action of such bioactivesubstances which target a specific protein or enzyme required by the particular virus. Alternatively, the bioactive-specific antiviral activity was neither examined nor evaluated against other viruses. This does not indicate a lack of activity, but rather a lack of experimental evidence.

Our analysis showed that several bioactive agents exhibited a broad spectrum of antiviral activities against both RNA and DNA viruses. For example, baicalin, sulforaphane, apigenin, ginkgolic acid, andrographolide, EGCG, resveratrol, berberine, quercetin, curcumin, and glycyrrhizic acid showed inhibitory effects on 5, 5, 9, 11, 12, 13, 13, 14, 17, and 18 distinct viruses, respectively.

Furthermore, we observed that many bioactive anti-viral compounds exhibited anti-viral activity against RNA viruses but had little or no effect against DNA viruses. This is especially evident in the case of sulforaphane, silvestrol, orsaponin, plitidepsin, and raoulic acid which inhibited only RNA viruses and had no impact on any of the DNA viruses evaluated. Similarly, the majority of viruses inhibited by apigenin and baicalein were RNA viruses. This may indicate that the above bioactive agents may directly or indirectly affect the enzymes found in RNA viruses such as RT or the RNA-dependent RNA polymerase (RdRp) activity.

Substances in natural products have shown epigenetic modifying activity and can affect the replication of various viruses. These plant-derived epigenetic modifiers alter gene expression by targeting the epigenome, reversible DNA/protein modification, and chromatin remodeling. Viruses rely on epigenome modification to ensure successful replication, latency, or escape from the immune response system. The reversal of the epigenome by plant-derived bioactive substances could result in blocking the virus’ life cycle, disrupting latency, and activating the immune response to fight viral infections.

The use of epigenetic modifiers as a means to combat viral infections has several advantages. As epigenetic modifications play an essential role in controlling viral gene expression, it is possible to block the replication and expression of a variety of viruses by targeting these modifications. This approach is applicable against both DNA and RNA viruses, making it a viable tool to combat a broad spectrum of viral diseases.

Because host epigenetic mechanisms are less susceptible to mutation, the likelihood of developing resistance is low, allowing for longer antiviral efficacy. Some viral infections, such as HSV and HIV, can lead to latent infections in host cells, making them difficult to eradicate. Epigenetic manipulations have the potential to reactivate dormant viruses so that they can be targeted and eliminated by the immune system or antiviral therapies. Focusing on epigenetic pathways may therefore offer a potential approach to overcome viral latency and eliminate the virus completely. Epigenetic therapies might be used in the future in conjunction with standard antiviral drugs or vaccines to increase their efficacy. By altering host factors, epigenetic therapies can create a hostile environment for viral replication and make viruses more susceptible to the direct antiviral effects of conventional drugs.

This synergy may improve therapeutic outcomes and minimize the possibility of treatment failure. The treatment of viral infections via epigenetic signaling pathways presents some unique difficulties. The mechanisms of epigenetic regulation are complicated and not fully understood. This makes it difficult to develop safe and effective drugs that target epigenetic mechanisms. In addition, epigenetic mechanisms control gene expression in all cells, not just those infected with viruses. This means that plant-based agents with epigenetic targets must focus on the epigenetic processes involved in viral replication.

Most natural products have been shown to be safe and well tolerated in animal studies, but further clinical studies are needed to confirm their safety and efficacy in humans. These natural products represent a promising new class of antiviral agents because they target different steps in the viral life cycle, reducing the likelihood of viruses developing resistance. Although there is no guarantee that natural products will be effective against all unknown viruses, it is likely that their ability to act on host functions will make them effective in combating future viral infections.

The bioavailability of natural products in humans is a complicated issue that is influenced by elements such as the properties of the compound, the route of administration, and individual physiology. The therapeutic potential of natural products is limited because they are often less bioavailable than synthetic drugs. However, there are many ways to improve the bioavailability of natural products, such as advanced delivery systems or focusing on specific tissues.

Further clinical studies are needed to confirm their safety and efficacy. Innovative in vitro and in vivo studies are among the avenues for exploring natural products with broad antiviral activity. The discovery of novel natural product derivatives with enhanced antiviral activity and improved bioavailability is a promising avenue of research. The development of innovative natural product delivery technologies, such as nanocarriers, could overcome the bioavailability issues faced by most plant-derived bioactive substances.

## Figures and Tables

**Table 3:** Anti-viral activity of plant bioactive substances.

Name	Structure	Anti-Viral Activity and Mode of Action
Andrographolide	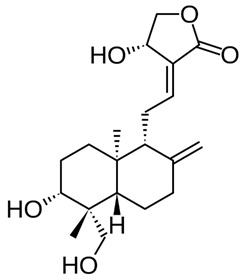	Modulates miR-377 to regulate HO-1. Downregulates miR-433 to regulate glutathione cysteine ligase. Upregulates miR-17, miR-224, and miR-181a [190]. Inhibits virus replications such HIV, HSV-1, HBV, HCV, ZIKV, DENV, CHIKV, FMDV, and IAV [181,182,184]. Prevents EBV reactivation by suppressing EBV lytic genes via histone modifications [186]. Also prevents IVA-induced inflammation through modulation of NF-κB and JAK-STAT signaling pathways [189].
Apigenin	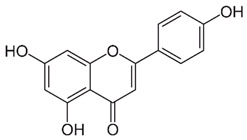	A potent inhibitor of HDAC1, 3 [192]. Exhibits antiviral properties against IAV, HRV, HSV, enterovirus, HBV, HCV, EV71, CVB1, and SARS-CoV-2 [195,196,197,198,200]. Anti-viral activity is attributed to the inhibition of HDAC activity and chromatin remodeling [192,199]. Downregulates miR-103 [201], miR-155 [111], and miR-122 [202] to affect HCV and HIV replication [113,114,115].
Baicalein	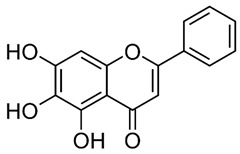	Inhibits HBV [204], HIV [205], DENV [206], and HSV-1 [207]. Inhibits DNMT and HDAC and affects influence epigenetic modifications [208,209]. Upregulates miR-3,178 [210] which targets HDAC10 [209]. Exhibits potent antiviral activity against DENV [206]
Berberine	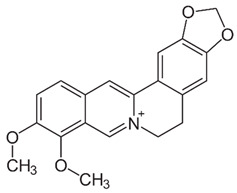	Regulates AMPK activity [212] and suppresses SIRT1 deacetylases [214]. Inhibits miR-21 expression [215]. Inhibits replication of HSV [217], HCMV [218], HPV [219], DENV [220], HIV [221], HCV [222], IAV [224], and SARS-CoV-2 [223]. Also inhibits p38 MAPK activity [39] which might explain its anti-viral activity [42,43,44,45,46,226].
Betulinic acid	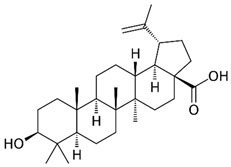	A computational approach predicted the capacity to alter HDAC6 and HDAC10 activity [229]. Inhibits DENV-2 NS5 polymerase activity [231] and HBV replication [232]. C-3 esterification of lead resulted in the discovery of Bevirimat, an HIV-1 maturation inhibitor.
Butyric acid	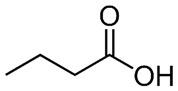	Induces HDAC expression and activity by upregulation of miR-203 promoter methylation [233]. Inhibits HBV replication by reducing HBx protein expression, HBV-DNA, and HBsAg [234].
Cardamonin	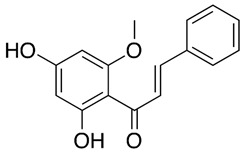	Exhibits antiviral action against the human coronavirus HCoV-OC43 which was mediated by induction of p38 MAPK signaling pathway [236].
Cordycepin	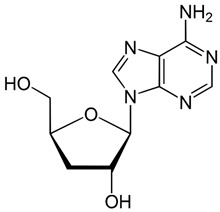	Promotes methylation of EBV genomic sites near Fp/Qp promoters. Increases DNMT3 [238] and reduces EBV replication [239]. As adenosine derivative, it exhibits antiviral activity against several viruses, including IAV, plant viruses, HIV, murine leukemia virus, EBV [241,242,243,244,245], and COVID-19 [246].
Corosolic acid	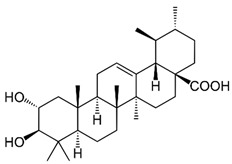	Alters CpG methylation sites, resulting in altered gene expression [249]. Increases the expression of acetylated histone H3 lysine 27 (H3K27ac), while decreasing histone H3 lysine 27 trimethylation (H3K27Me3) [251]. Exhibits anti-viral activity against a number of viruses [248].
Curcumin	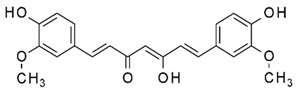	Decreases the expression of HDAC1, HDAC3, HDAC8, and histone acetyltransferase p300 while enhancing the expression of Ac-histone H4 protein [253]. Reduces HAT activity and inhibits DNMT [254]. Inhibition of HBV replication was attributed to a decrease in the acetylation level of cccDNA-bound histone H3 and H4 [255]. Downregulates miR-350, miR-17-2-3p, let 7e-3p, miR-1224, miR-466b-1-3p, miR-18a-5p, and miR-322-5p. Upregulates miR-122-5p, miR-3473, miR-182, and miR-344a-3p [256]. Inhibits the replication of various viruses, including HBV [255], HIV [258], IAV [259], HPV-18 [261], ZIKV, CHIKV, VSV, CVB3, EV71, RSV, HSV-2, KSHV, and HAdV [263].
Ellagic acid	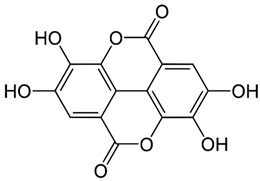	Increases HDACs’ gene expression and histone arginine methylation [268]. Decreases H3K9 acetylation and HDAC9 dissociation [268]. Inhibits SARS-CoV-2 viral entry and replication [269].
Epigallocatechin gallate (EGCG)	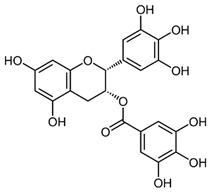	Inhibits the activity of DNMT 1, DNMT 3a, DNMT 3b [273], and HDACs [275] and downregulates the expression of HDAC1, HDAC2, and HDAC3 [277]. Inhibits HAT activity [278]. Decreases the levels of let-7e-5p, miR-103a-3p, miR-151a-5p, miR-195-5p, miR-222-3p, miR-23a-3p, miR-23b-3p, miR-26a-5p, miR-27a-3p, miR-29b-3p, miR-3195, miR-3651, miR-4281, miR-4459, miR-4516, miR-762, and miR-125b-5p [283]. Induces the expression of miR-3663-3p, miR-1181, miR-3613-3p, miR1281, miR-1539, miR-221-5p, miR-374b, miR-4306, miR-500a-5p, miR590-5p miR-140-3p, and miR-221 [284,285,286]. Inhibits the replication of IAV, HBV, HCV, HSV-1 and HSV-2, HPV, ZIKV, and SARS-CoV-2 [288,289,290,291,292,293,294]. Upregulates miR-548m and inhibits miR-122 expression, which modulates HCV infectivity [291]. Upregulates let-7 to increase interferon expression and inhibit IAV infection [202].
Galangin	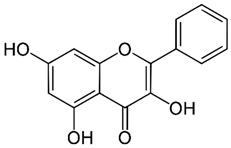	Inhibits HDAC activity [299] and upregulates miR-455-5p [300]. Exhibits antiviral activity against HSV-1 and CoxB1 [301].
Garcinol	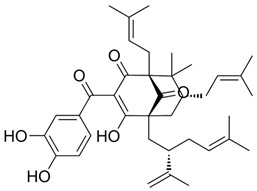	Decreases HAT activity of p300 and pCAF [303]. Downregulates miR-21, miR-494, miR-495, and miR-1977 [309]. Upregulates miR-453, miR-128, miR-1280 and miR-720, let-7a, let-7e, let-7f, miR-200b, and miR-200c [311]. Inhibits HIV-1 reverse-transcriptase-associated ribonuclease H [312].
Genistein	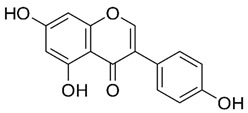	Reduces HDAC while increasing HAT activity [313]. Inhibits miR-223 and miR-223 expression [316] which is involved in regulation of immune response and viral infections [115,116,117].
Ginkgolic acid	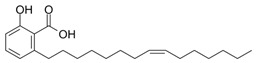	Impairs protein SUMOylation [318]. Inhibits HSV-1, HSV-2, VZV, HCMV, ZIKV, IAV, EBV, HIV, EBOV, and Coronavirus COVID-19 [342].
Glycyrrhizic acid	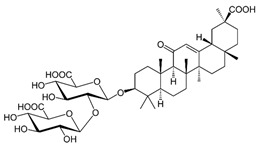	Inhibits replications of various viruses including HBV, HCV, IAV H1N1, HIV [326], NDV [327], SARS-CoV-2 [458,459,460], RSV, VACV, HSV [329], and VSV [329,330]. Exhibits anti-inflammatory effects, decreasing IL-6 release [331] by regulating NF-κB and PI3K signaling pathways [333]. Inhibits viral replication of various viruses including HBV, HCV, IAV H1N1, and HIV [326].
Grifolin	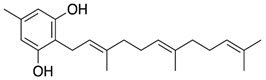	Reduces Elk1 transcription as well as its binding to the DNMT1 promoter region [461]. Modulates ERK1/2-Elk1-DNMT1 signaling [344].
Oleacein	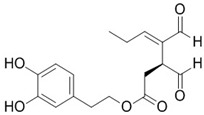	Downregulates several class I/II HDACs [346,347]. Exhibits antiviral effect against HIV-1 [349].
Plitidepsin	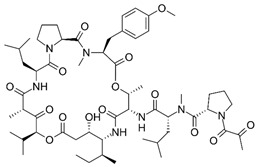	Targets the eukaryotic translation elongation factor 1A (eEF1A) [382]. Exhibits anti-viral activity against RSV, gastroenteritis coronavirus [383], and SARS-CoV-2 [384,385].
Pterostilbene	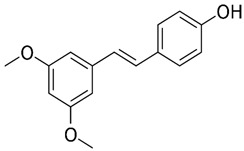	Modulates HDAC activity and inhibits SIRT1 [387,388]. Inhibits SARS-CoV-2 replication [389]
Quercetin	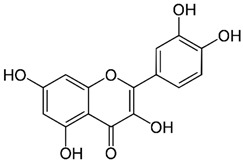	Enhances histone H3 acetylation, activates HAT, and inhibits HDAC activities [391]. Inhibits HMT [213]. Inhibits miR-146a expression [392], a regulator of HIV replication [393], and miR-16, miR-217, and miR-145 [395,396,397]. Inhibits replication of IAV H1N1, IVA H3N2, HBV, HCV, DENV, poliovirus, rhinovirus, CHIKV, MERS-CoV, HSV 1/2, EBV, RSV, Arbovirus, EBOV, HIV, Japanese encephalitis virus, hAdV, enterovirus, ZIKV, NDV, MAYV, and SARS-CoV-2 [197,293,398,399,400]. Activates SIRT1 which resulted in inhibition of HCV [403] Upregulates let-7 which restores anti-viral immune response and thus exhibits anti-IVA activity [202].
Resveratrol	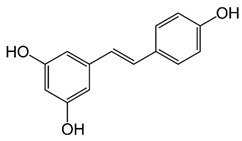	Inhibits HDAC [406,407] and activates SIRT1 [409]. Decreases the levels of miR-17, miR-21, miR-25, miR-92a-2, miR-103-1, and miR-103-2 [410] and upregulates miR-200c [411]. In a human study, it increased miR-21, miR-181b, miR-663, and miR-30c, while reducing inflammatory cytokines like IL-6, CCL3, IL-1β, and TNF-α [412]. Inhibits HSV infection [414], beta-corona viruses such as MERS-COV and SARS-CoV-2 [415], Varicella-zoster virus (VZV) wild-type and DNA polymerase mutants with acyclovir-resistant VZV [419,420], VEEV [421], EBV [422], CV [423], and RSV. SIRT proteins regulate HBV replication and thus SIRT modulators such as resveratrol are suitable as to be used against HBV and RSV infections [402,425].
Silibinin	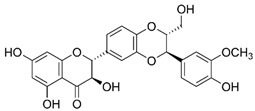	Inhibits the expression of HDAC1, HDAC2, HDAC3, HDAC6, SET domain proteins (SETD1A, D4, D6), and lysine-specific demethylases (KDM 5B, 5C, 4A) [315]. Inhibits DNMTs [428,429,430]. Downregulates expression of miR-21 and miR-155 [431]. Exhibits anti-viral activity against HBV, DENV, CHIKV, MAYV, IVA, HIV, and HBV [432].
Silvestrol	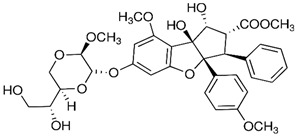	Targets the eukaryotic initiation factor-4A (eIF4A) [434]. Exhibits activity against EBOV, ZIKV, CHIKV and coronaviruses, MERS-CoV, HCoV-229E, and SARS-CoV-2 [435,436,437,438,439,440].
Sulforaphane	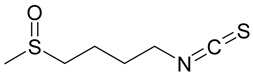	Reduces HDAC activity [442] while increases the expression of acetylated histones H3 and H4 [442]. Upregulates let-7 expression, exhibits anti-IVA activity [202], and diminishes viral-induced immune cell activation in the lungs [446]. Inhibits replications of HCV and DENV [444]
Tanshinone IIA	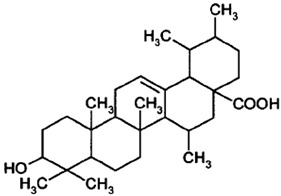	Decreases the expression and activity of HDACs [449]. Inhibits MAPK p38 which resulted in, lowering the replications of a number of viruses including DENV [43], coronavirus [44], VEEV [45], EV71 [46], SFTSV, HSV-1, and SARS-CoV-2 [47,450].
Ursolic acid	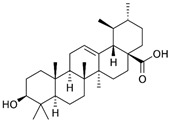	Reduces the expression of HDAC1, HDAC2, HDAC3, HDAC8 (Class I), HDAC6, and HDAC7 (Class II) [452]. Exhibits anti-CMV activity [453].
Withaferin A	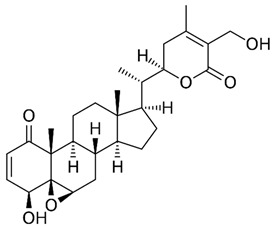	Downregulates HDAC1 [454,455]. Decreases HMT activity, but enhances HAT activity [455]. Inhibits Mpro main protease of SARS-CoV-2 [456]. Attenuates H1N1 IVA [457]
